# A polynomial time biclustering algorithm for finding approximate expression patterns in gene expression time series

**DOI:** 10.1186/1748-7188-4-8

**Published:** 2009-06-04

**Authors:** Sara C Madeira, Arlindo L Oliveira

**Affiliations:** 1 Knowledge Discovery and Bioinformatics (KDBIO) group, INESC-ID, Lisbon, Portugal; 2 Instituto Superior Técnico, Technical University of Lisbon, Lisbon, Portugal; 3 University of Beira Interior, Covilhã, Portugal

## Abstract

**Background:**

The ability to monitor the change in expression patterns over time, and to observe the emergence of coherent temporal responses using gene expression time series, obtained from microarray experiments, is critical to advance our understanding of complex biological processes. In this context, biclustering algorithms have been recognized as an important tool for the discovery of local expression patterns, which are crucial to unravel potential regulatory mechanisms. Although most formulations of the biclustering problem are NP-hard, when working with time series expression data the interesting biclusters can be restricted to those with contiguous columns. This restriction leads to a tractable problem and enables the design of efficient biclustering algorithms able to identify all maximal contiguous column coherent biclusters.

**Methods:**

In this work, we propose *e*-CCC-Biclustering, a biclustering algorithm that finds and reports all maximal contiguous column coherent biclusters with approximate expression patterns in time polynomial in the size of the time series gene expression matrix. This polynomial time complexity is achieved by manipulating a discretized version of the original matrix using efficient string processing techniques. We also propose extensions to deal with missing values, discover anticorrelated and scaled expression patterns, and different ways to compute the errors allowed in the expression patterns. We propose a scoring criterion combining the statistical significance of expression patterns with a similarity measure between overlapping biclusters.

**Results:**

We present results in real data showing the effectiveness of *e*-CCC-Biclustering and its relevance in the discovery of regulatory modules describing the transcriptomic expression patterns occurring in *Saccharomyces cerevisiae *in response to heat stress. In particular, the results show the advantage of considering approximate patterns when compared to state of the art methods that require exact matching of gene expression time series.

**Discussion:**

The identification of co-regulated genes, involved in specific biological processes, remains one of the main avenues open to researchers studying gene regulatory networks. The ability of the proposed methodology to efficiently identify sets of genes with similar expression patterns is shown to be instrumental in the discovery of relevant biological phenomena, leading to more convincing evidence of specific regulatory mechanisms.

**Availability:**

A prototype implementation of the algorithm coded in Java together with the dataset and examples used in the paper is available in .

## Background

Time series gene expression data, obtained from microarray experiments performed in successive instants of time, can be used to study a wide range of biological problems [1], and to unravel the mechanistic drivers characterizing cellular responses [2]. Being able to monitor the change in expression patterns over time, and to observe the emergence of coherent temporal responses of many interacting components, should provide the basis for understanding evolving but complex biological processes, such as disease progression, growth, development, and drug responses [2]. In this context, several machine learning methods have been used in the analysis of gene expression data [3]. Recently, biclustering [4-6], a non-supervised approach that performs simultaneous clustering on the gene and condition dimensions of the gene expression matrix, has been shown to be remarkably effective in a variety of applications. The advantages of biclustering in the discovery of local expression patterns, described by a coherent behavior of a subset of genes in a subset of the conditions under study, have been extensively studied and documented [4-8]. Recently, Androulakis et al. [2] have emphasized the fact that biclustering methods hold a tremendous promise as more systemic perturbations are becoming available and the need to develop consistent representations across multiple conditions is required. Madeira et al. [9] have also described the use of biclustering as critical to identify the dynamics of biological systems as well as the different groups of genes involved in each biological process. However, most formulations of the biclustering problem are NP-hard [10], and almost all the approaches presented to date are heuristic, and for this reason, not guaranteed to find optimal solutions [6]. In a few cases, exhaustive search methods have been used [7,11], but limits are imposed on the size of the biclusters that can be found [7] or on the size of the dataset to be analyzed [11], in order to obtain reasonable runtimes. Furthermore, the inherent difficulty of this problem when dealing with the original real-valued expression matrix and the great interest in finding coherent behaviors regardless of the exact numeric values in the matrix, has led many authors to a formulation based on a discretized version of the expression matrix [7-9,12-23]. Unfortunately, the discretized versions of the biclustering problem remain, in general, NP-hard. Nevertheless, in the case of time series expression data the interesting biclusters can be restricted to those with contiguous columns leading to a tractable problem. The key observation is the fact that biological processes are active in a contiguous period of time, leading to increased (or decreased) activity of sets of genes that can be identified as biclusters with contiguous columns. This fact led several authors to point out the relevance of biclusters with contiguous columns and their importance in the identification of regulatory mechanisms [9,20,22,24].

In this work, we propose *e*-CCC-Biclustering, a biclustering algorithm specifically developed for time series expression data analysis, that finds and reports all maximal contiguous column coherent biclusters with approximate expression patterns in time polynomial in the size of the expression matrix. The polynomial time complexity is obtained by manipulating a discretized version of the original expression matrix and by using efficient string processing techniques based on suffix trees. These approximate patterns allow a given number of errors, per gene, relatively to an expression profile representing the expression pattern in the bicluster. We also propose several extensions to the core *e*-CCC-Biclustering algorithm. These extensions improve the ability of the algorithm to discover other relevant expression patterns by being able to deal with missing values directly in the algorithm and by taking into consideration the possible existence of anticorrelated and scaled expression patterns. Different ways to compute the errors allowed in the approximate patterns (restricted errors, alphabet range weighted errors and pattern length adaptive errors) can also be used. Finally, we propose a statistical test that can be used to score the biclusters discovered (by extending the concept of statistical significance of an expression pattern [9] to cope with approximate expression patterns) and a method to filter highly overlapping, and, therefore, redundant, biclusters. We report results in real data showing the effectiveness of the approach and its relevance in the process of identifying regulatory modules describing the transcriptomic expression patterns occurring in *Saccharomyces cerevisiae *in response to heat stress. We also show the superiority of *e*-CCC-Biclustering when compared with state of the art biclustering algorithms, specially developed for time series gene expression data analysis such as CCC-Biclustering [9,22].

### Related Work: Biclustering algorithms for time series gene expression data

Although many algorithms have been proposed to address the general problem of biclustering [5,6], and despite the known importance of discovering local temporal patterns of expression, to our knowledge, only a few recent proposals have addressed this problem in the specific case of time series expression data [9,20,22,24]. These approaches fall into one of the following two classes of algorithms:

1. Exhaustive enumeration: CCC-Biclustering [9,22] and *q*-clustering [20].

2. Greedy iterative search: CC-TSB algorithm [24].

These three biclustering approaches work with a single time series expression matrix and aim at finding biclusters defined as subsets of genes and subsets of **contiguous** time points with coherent expression patterns. CCC-Biclustering and *q*-clustering work with a discretized version of the expression matrix while the CC-TSB-algorithm works with the original real-valued expression matrix. In additional file 1: related_work we describe in detail these algorithms and identify their strengths and weaknesses. Based on their characteristics, we decided to compare the performance of *e*-CCC-Biclustering with that of CCC-Biclustering, but not with that of the *q*-clustering and CC-TSB algorithms. The decision to exclude the last two algorithms from the comparisons is mainly based on existing analysis of these algorithms [9], and is basically related with complexity issues, in the case of *q*-clustering, and on poor results on real data obtained by the heuristic approach used by the CC-TSB algorithm.

### Biclusters in discretized gene expression data

**Figure 1:**
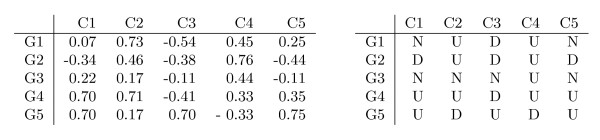
**Illustrative example of the discretization process**. This figure shows: **(Left) **Original expression matrix *A'*; and **(Right) **Discretized matrix *A *obtained by considering a simple discretization technique, which uses a three symbol alphabet Σ = {*D*, *N*, *U*}. The symbols mean down-regulation (*D*), up-regulation (*U*) or no-change (*N*). In this case, the values  ∈ ]-0.3, 0.3[ were discretized to *N*, and the values  ≤ -0.3 and  ≥ 0.3 were discretized to *D *and *U*, respectively.

Let *A' *be an |*R*| row by |*C*| column gene expression matrix defined by its set of rows (genes), *R*, and its set of columns (conditions), *C*. In this context,  represents the expression level of gene *i *under condition *j*. In this work, we address the case where the gene expression levels in matrix *A' *can be discretized to a set of symbols of interest, Σ, that represent distinctive activation levels. After the discretization process, matrix *A' *is transformed into matrix *A*, where *A*_*ij *_∈ Σ represents the discretized value of the expression level of gene *i *under condition *j *(see Figure 1 for an illustrative example).

Given matrix *A *we define the concept of bicluster and the goal of biclustering as follows:

**Definition 1 (Bicluster) ***A bicluster is a sub-matrix A*_*IJ *_*defined by I *⊆ *R, a subset of rows, and J *⊆ *C, a subset of columns. A bicluster with only one row or one column is called trivial*.

The goal of biclustering algorithms is to identify a set of biclusters *B*_*k *_= (*I*_*k*_, *J*_*k*_) such that each bicluster satisfies specific characteristics of homogeneity. These characteristics vary in different applications [6]. In this work we will deal with biclusters that exhibit coherent evolutions:

**Definition 2 (CC-Bicluster) ***A column coherent bicluster A*_*IJ *_*is a bicluster such that A*_*ij *_= *A*_*lj *_*for all rows i, l *∈ *I and columns j *∈ *J*.

Finding all maximal biclusters satisfying this coherence property is known to be an NP-hard problem [10].

### CC-Biclusters in discretized gene expression time series

Since we are interested in the analysis of time series expression data, we can restrict the attention to potentially overlapping biclusters with arbitrary rows and contiguous columns [9,20,22,24]. This fact leads to an important complexity reduction and transforms this particular version of the biclustering problem into a tractable problem. Previous work in this area [9,22] has defined the concept of CC-Biclusters in time series expression data and the important notion of maximality:

**Definition 3 (CCC-Bicluster) ***A contiguous column coherent bicluster A*_*IJ *_*is a subset of rows I *= {*i*_1_, ..., *i*_*k*_} *and a subset of ****contiguous ****columns J *= {*r*, *r *+ 1, ..., *s *- 1, *s*} *such that A*_*ij *_= *A*_*lj*_, *for all rows i*, *l *∈ *I and columns j *∈ *J. Each CCC-Bicluster defines a string S that is common to every row in I for the columns in J*.

**Definition 4 (row-maximal CCC-Bicluster) ***A CCC-Bicluster A*_*IJ *_*is row-maximal if we cannot add more rows to I and maintain the coherence property referred in Definition 3*.

**Definition 5 (left-maximal and right-maximal CCC-Bicluster) ***A CCC-Bicluster A*_*IJ *_*is left-maximal/right-maximal if we cannot extend its expression pattern S to the left/right by adding a symbol (contiguous column) at its beginning/end without changing its set of rows I*.

**Definition 6 (maximal CCC-Bicluster) ***A CCC-Bicluster A*_*IJ *_*is maximal if no other CCC-Bicluster exists that properly contains A*_*IJ*_, *that is*, *if for all other CCC-Biclusters A*_*LM*_, *I *⊆ *L *∧ *J *⊆ *M *⇒ *I *= *L *∧ *J *= *M*.

**Lemma 1 ***Every maximal CCC-Bicluster is right, left and row-maximal*.

**Figure 2:**
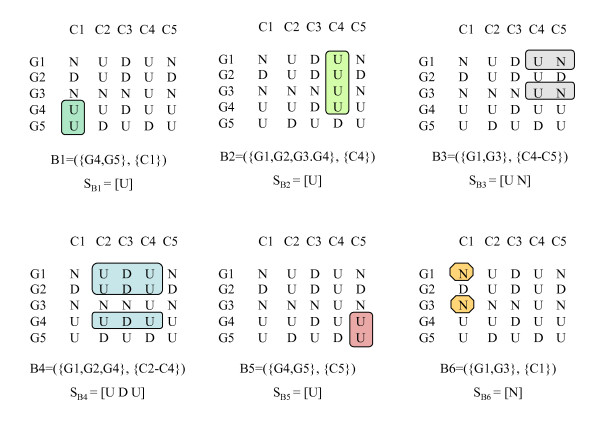
**Maximal CCC-Biclusters in a discretized matrix**. This figure shows all maximal CCC-Biclusters with at least two rows that can be identified in the discretized matrix in Figure 1. The strings *S*_*B*1 _= [*U*], *S*_*B*2 _= [*U*], *S*_*B*3 _= [*UN*], *S*_*B*4 _= [*UDU*], *S*_*B*5 _= [*U*] and *S*_*B*6 _= [*N*] correspond to the expression patterns of the maximal CCC-Biclusters identified as B1, B2, B3, B4, B5 and B6, respectively.

Figure 2 shows the maximal CCC-Biclusters with at least two rows (genes) present in the discretized matrix in Figure 1. CCC-Biclusters with only one row, even when maximal, are trivial and uninteresting from a biological point of view and are thus discarded.

### Maximal CCC-Biclusters and generalized suffix trees

**Figure 3:**
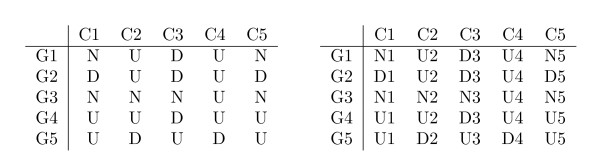
**Illustrative example of the alphabet transformation performed after the discretization process**. This figure shows: **(Left) **Discretized matrix *A *in Figure 1; **(Right) **Discretized matrix *A *after alphabet transformation.

**Figure 4:**
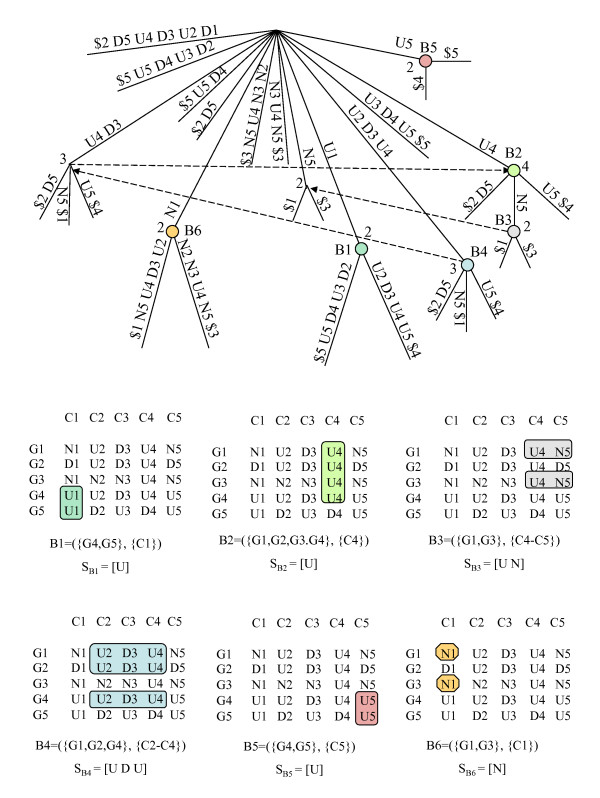
**Maximal CCC-Biclusters and generalized suffix trees**. This figure shows: **(Top) **Generalized suffix tree constructed for the transformed matrix in Figure 3. For clarity, this figure does not contain the leaves that represent string terminators that are direct daughters of the root. Each internal node, other than the root, is labeled with the number of leaves in its subtree. We show the suffix links between nodes although (for clarity) we omit the suffix links pointing to the root. All maximal CCC-Biclusters are identified using a circle. The labels B1 to B6 identify the nodes corresponding to all maximal CCC-Biclusters with at least two rows/genes. Note that the rows in each CCC-Bicluster identified by a given node *v *are obtained from the string terminators in its subtree. The value of the string-depth of *v *and the first symbol in the string-label of *v *provide the information needed to identify the set of contiguous columns. **(Bottom) **Maximal CCC-Biclusters B1 to B6 showed in the discretized matrix as subsets of rows and columns. The strings *S*_*B*1 _= [U], *S*_*B*2 _= [U], *S*_*B*3 _= [U N], *S*_*B*4 _= [U D U], *S*_*B*5 _= [U] and *S*_*B*6 _= [N] correspond to the expression patterns of the maximal CCC-Biclusters identified as B1 to B6, respectively.

Consider the discretized matrix *A *obtained from matrix *A' *using the alphabet Σ. Consider also the matrix obtained by preprocessing *A *using a simple alphabet transformation, that appends the column number to each symbol in the matrix (see Figure 3), and considers a new alphabet Σ*' *= Σ × {1, ..., |*C*|}, where each element Σ*' *is obtained by concatenating one symbol in Σ and one number in the range {1, ..., |*C*|}. We present below the two Lemmas and the Theorem describing the relation between maximal CCC-Biclusters with at least two rows and nodes in the generalized suffix tree built from the set of strings obtained after alphabet transformation [9,22]. Figure 4 illustrates this relation using the generalized suffix tree obtained from the rows in the discretized matrix after alphabet transformation in Figure 3 together with the maximal CCC-Biclusters with at least two rows (B1 to B6) already showed in Figure 2.

**Lemma 2 ***Every right-maximal, row-maximal CCC-Bicluster with at least two rows corresponds to one internal node in T and every internal node in T corresponds to one right-maximal, row-maximal CCC-Bicluster with at least two rows*.

**Lemma 3 ***An internal node in T corresponds to a left-maximal CCC-Bicluster iff it is a MaxNode*.

**Definition 7 (MaxNode) ***An internal node v in T is called a MaxNode iff it satisfies one of the following conditions:*

*a) It does not have incoming suffix links*.

*b) It has incoming suffix links only from nodes u_i _such that, for every node u_i_, the number of leaves in the subtree rooted at u_i _is inferior to the number of leaves in the subtree rooted at v*.

**Theorem 1 ***Every maximal CCC-Bicluster with at least two rows corresponds to a MaxNode in the generalized suffix tree T, and each MaxNode defines a maximal CCC-Bicluster with at least two rows*.

Note that this theorem is the base of CCC-Biclustering [9,22], which finds and reports all maximal CCC-Biclusters using three main steps:

1. All internal nodes in the generalized suffix tree are marked as "*Valid"*, meaning each of them identifies a row-maximal, right-maximal CCC-Bicluster with at least two nodes according to Lemma 2.

2. All internal nodes identifying non left-maximal CCC-Biclusters are marked as "*Invalid" *using Theorem 1, discarding all row-maximal, right-maximal CCC-Biclusters which are not left-maximal.

3. All maximal CCC-Biclusters, identified by each node marked as "*Valid"*, are reported.

## Methods

In this section we propose *e*-CCC-Biclustering, an algorithm designed to find and report all maximal CCC-Biclusters with approximate expression patterns (*e*-CCC-Biclusters) using a discretized matrix *A *and efficient string processing techniques. We first define the concepts of *e*-CCC-Bicluster and maximal *e*-CCC-Bicluster. We then formulate two problems: (1) finding all maximal *e*-CCC-Biclusters and (2) finding all maximal *e*-CCC-Biclusters satisfying row and column quorum constraints. We discuss the relation between maximal *e*-CCC-Biclusters and generalized suffix trees highlighting the differences between this relation and that of maximal CCC-Biclusters and generalized suffix tree, discussed in the previous section. We then discuss and explore the relation between the two problems above and the *Common Motifs Problem *[25,26]. We describe *e*-CCC-Biclustering, a polynomial time algorithm designed to solve both problems and sketch the analysis of its computational complexity. We present extensions to handle missing values, discover anticorrelated and scaled expression patterns, and consider alternative ways to compute approximate expression patterns. Finally, we propose a scoring criterion for *e*-CCC-Biclusters combining the statistical significance of their expression patterns with a similarity measure between overlapping biclusters.

### CCC-Biclusters with approximate expression patterns

The CCC-Biclusters defined in the previous section are *perfect*, in the sense that they do not allow errors in the expression pattern *S *that defines the CCC-Bicluster. This means that all genes in *I *share *exactly *the same expression pattern in the time points in *J*. Being able to find all maximal CCC-Biclusters using efficient algorithms is useful to identify potentially interesting expression patterns and can be used to discover regulatory modules [9]. However, some genes might not be included in a CCC-Bicluster of interest due to errors. These errors may be measurement errors, inherent to microarray experiments, or discretization errors, introduced by poor choice of discretization thresholds or inadequate number of discretization symbols. In this context, we are interested in CCC-Biclusters with *approximate *expression patterns, that is, biclusters where a certain number of errors is allowed in the expression pattern *S *that defines the CCC-Bicluster. We introduce here the definitions of *e*-CCC-Bicluster and maximal *e*-CCC-Bicluster preceded by the notion of *e*-neighborhood:

**Definition 8 (***e***-Neighborhood) ***The e-Neighborhood of a string S of length |S|, defined over the alphabet Σ with |Σ| symbols, N*(*e*, *S*), *is the set of strings S*_*i*_, *such that*: |*S*| = |*S*_*i*_| *and Hamming*(*S*, *S*_*i*_) ≤ *e, where e is an integer such that e *≥ 0. *This means that the Hamming distance between S and S*_*i *_*is no more than e, that is, we need at most e symbol substitutions to obtain S*_*i *_*from S*.

**Lemma 4 ***The e-Neighborhood of a string S, N*(*e*, *S*), *contains **elements*.

**Definition 9 (***e***-CCC-Bicluster) ***A contiguous column coherent bicluster with e errors **per gene***, *e-CCC-Bicluster, is a CCC-Bicluster A*_*IJ *_*where all the strings S*_*i *_*that define the expression pattern of each of the genes in I are in the e-Neighborhood of an expression pattern S that defines the e-CCC-Bicluster: S*_*i *_∈ *N *(*e*, *S*), ∀*i *∈ *I. The definition of 0-CCC-Bicluster is equivalent to that of a CCC-Bicluster*.

**Definition 10 (maximal ***e***-CCC-Bicluster) ***An e-CCC-Bicluster A*_*IJ *_*is maximal if it is row-maximal, left-maximal and right-maximal. This means that no more rows or contiguous columns can be added to I or J, respectively, maintaining the coherence property in Definition 9*.

Given these definitions we can now formulate the problem we solve in this work:

**Problem 1 **Given a discretized expression matrix *A *and the integer *e *≥ 0 identify and report all maximal *e*-CCC-Biclusters .

Similarly to what happened with CCC-Biclusters, *e*-CCC-Biclusters with only one row should be overlooked. A similar problem is that of finding and reporting *only *the maximal *e*-CCC-Biclusters satisfying predefined row and column quorum constraints:

**Problem 2 **Given a discretized expression matrix *A *and three integers *e ≥ *0, *q*_*r *_≥ 2 and *q*_*c *_≥ 1, where *q*_*r *_is the row quorum (minimum number of rows in *I*_*k*_) and *q*_*c *_is the column quorum (minimum number of columns in *J*_*k*_), identify and report all maximal *e*-CCC-Biclusters  such that, *I*_*k *_and *J*_*k *_have at least *q*_*r *_rows and *q*_*c *_columns, respectively.

**Figure 5:**
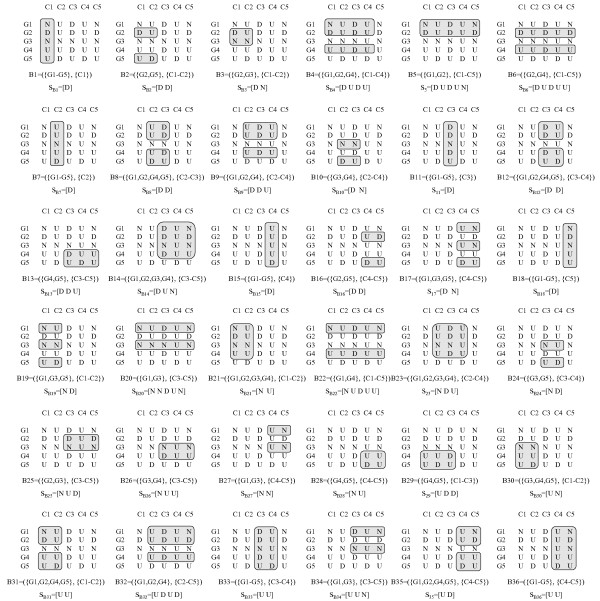
**Maximal *e*-CCC-Biclusters in a discretized matrix**. This figure shows all maximal 1-CCC-Biclusters with at least two rows that can be identified in the discretized matrix in Figure 1. Note that several of these 1-CCC-Biclusters can be defined by more than one expression pattern. For example, B1 can be defined by *S*_*B*1 _= [D], as shown in the figure, but can also be defined by *S*_*B*1 _= [N] or *S*_*B*1 _= [U]. Other 1-CCC-Biclusters are defined by expression patterns not occurring in the discretized matrix in the contiguous columns identifying the biclusters. This is the case of 1-CCC-Bicluster B2, for example, defined by the pattern *S*_*B*2 _= [D D], which does not occur in the columns C1–C2.

**Figure 6:**
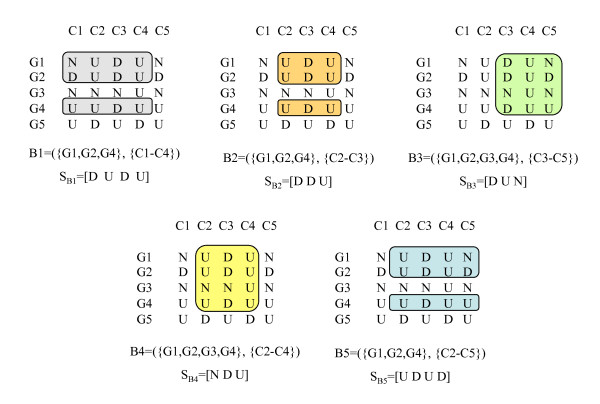
**Maximal *e*-CCC-Biclusters with row and column quorum constraints in a discretized matrix**. This figure shows the five maximal 1-CCC-Biclusters with at least 3 rows/columns (*q*_*r *_= *q*_*c *_= 3) that can be identified in the discretized matrix in Figure 1. These 1-CCC-Biclusters are defined, respectively, by the following patterns: *S*_*B*1 _= [D U D U], *S*_*B*2 _= [D D U], *S*_*B*3 _= [D U N], *S*_*B*4 _= [N D U] and *S*_*B*5 _= [U D U D]. Also clear from this figure is the fact that the same *e*-CCC-Bicluster can be defined by several patterns. For example, 1-CCC-Bicluster B1 can also be identified by the patterns [N U D U] and [U U D U]. An interesting example is the case of 1-CCC-Bicluster B2, which can also be defined by the patterns [N D U], [U N U], [U U U], [U D D] and [U D N]. Note however, that B2 cannot be identified by the pattern [U D U]. If this was the case, B2 would not be right maximal, since the pattern [U D N] can be extended to the right by allowing one error at column 5. In fact, this leads to the discovery of the maximal 1-CCC-Bicluster B5. Moreover, *e*-CCC-Biclusters can be defined by expression patterns not occurring in the discretized matrix. This is the case of 1-CCC-Biclusters B2 and B4, defined respectively by the patterns *S*_*B*2 _= [D D U] and *S*_*B*4 _= [N D U], which do not occur in the matrix in the contiguous columns defining B2 and B4 (C2–C3 and C2–C4, respectively).

Figure 5 shows all maximal *e*-CCC-Biclusters with at least rows (genes), which are present in the discretized matrix in Figure 1, when one error per gene is allowed (*e *= 1). Figure 6 shows all maximal *e*-CCC-Biclusters identified using row and column constraints. In this case, the maximal 1-CCC-Biclusters having at least three rows and three columns (*q*_*r *_= *q*_*c *_= 3) are shown. Also clear in these figures is the fact that, when errors are allowed (*e *> 0), different expression patterns *S *can define the same *e*-CCC-Bicluster. Furthermore, when *e *> 0, an *e*-CCC-Bicluster can be defined by an expression pattern *S*, which does not occur in the discretized matrix in the set of contiguous columns in the *e*-CCC-Bicluster.

### Maximal *e*-CCC-Biclusters and generalized suffix trees

In the previous section we showed that each internal node in the generalized suffix tree, constructed for the set of strings corresponding to the rows in the discretized matrix after alphabet transformation, identifies exactly one CCC-Bicluster with at least two rows (maximal or not) (see Lemma 2). We also showed that each internal node corresponding to a *MaxNode *(see Definition 7) in the generalized suffix tree identifies exactly *one *maximal CCC-Bicluster and that each maximal CCC-Bicluster is identified by exactly *one MaxNode *(see Lemma 3 and Theorem 1). This also implies that a maximal CCC-Bicluster is identified by *one *expression pattern, which is common to all genes in the CCC-Bicluster within the contiguous columns in the bicluster. Moreover, all expression patterns identifying maximal CCC-Biclusters *always occur *in the discretized matrix and thus correspond to a node in the generalized suffix tree (see Figure 4).

**Figure 7:**
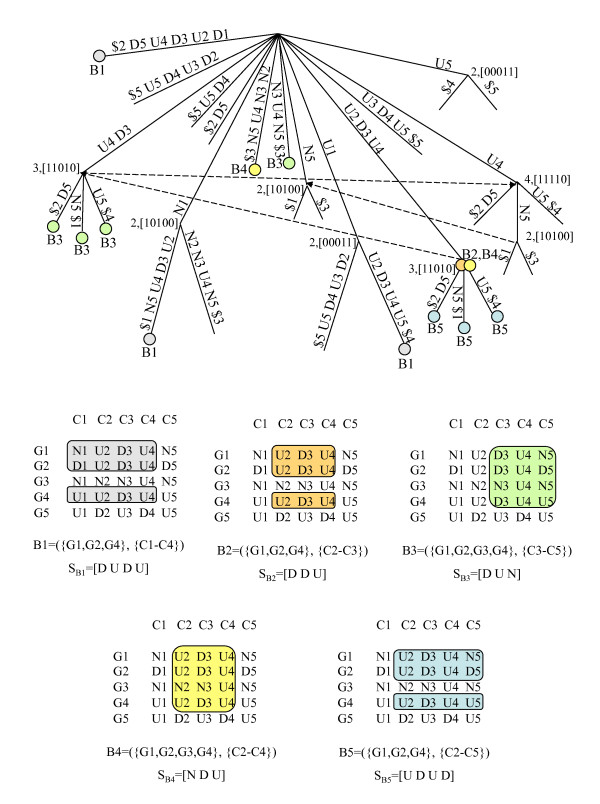
***e*-CCC-Biclusters (*e *> 0) and generalized suffix trees**. This figure shows: **(Top) **Generalized suffix tree constructed for the transformed matrix in Figure 3 (the information stored in the nodes correspond to the number of leaves and row identifiers in their subtree and is used by *e*-CCC-Biclustering). The circles labeled with B1, B2, B3, B4 and B5 identify the nodes related with the five maximal 1-CCC-Biclusters discovered when *e *= 1 and *q*_*e *_= *q*_*c *_= 3, shown in Figure 6; **(Bottom) **Maximal 1-CCC-Biclusters B1 to B5 showed in the matrix as subsets of rows and columns. The strings *S*_*B*1 _= [D U D U], *S*_*B*2 _= [D D U], *S*_*B*3 _= [D U N], *S*_*B*4 _= [N D U] and *S*_*B*5 _= [U D U D] correspond to the expression patterns defining the maximal 1-CCC-Biclusters identified as B1 to B5, respectively. Note that *e*-CCC-Biclusters can now be identified (and generally are) by more than one node in the generalized suffix tree. This is the case of 1-CCC-Biclusters B1, B3, B4 and B5. In fact only B2 is identified by a single node in this example. Moreover, a node in the generalized suffix tree might be related with more than one maximal *e*-CCC-Bicluster. Look for example at the node identifying approximate patterns occurring in both 1-CCC-Biclusters B2 and B4.

When errors are allowed, *one e*-CCC-Bicluster (*e > *0) can be identified (and usually is) by *several *nodes in the generalized suffix tree, constructed for the set of strings corresponding to the rows in the discretized matrix after alphabet transformation, and *one *node in the generalized suffix tree may be related with *multiple e*-CCC-Biclusters (maximal or not) (see Figure 7). Moreover, a maximal *e*-CCC-Bicluster can be defined by *several *expression patterns (see Figure 5 and Figure 6). Upon all this, a maximal *e*-CCC-Bicluster can be defined by an expression pattern *not occurring *in the expression matrix and thus not appearing in the generalized suffix tree (see Figure 6 and Figure 7).

**Figure 8:**
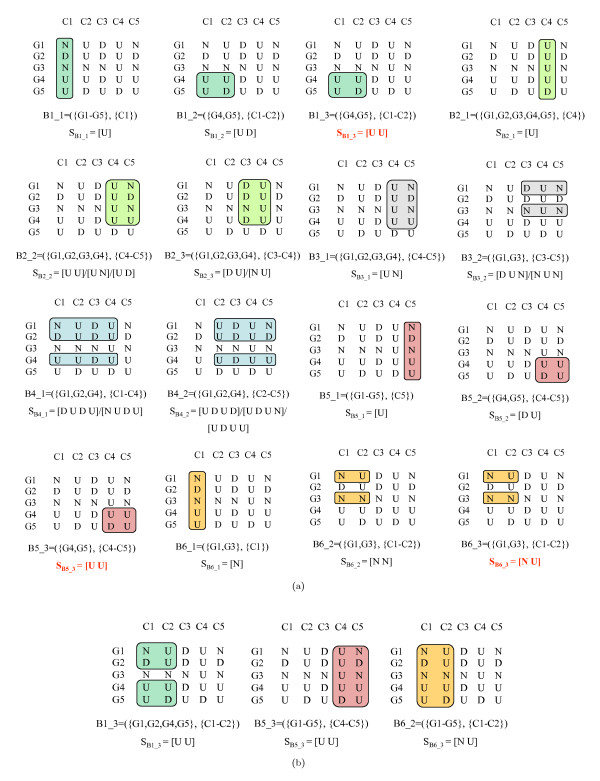
**Maximal CCC-Biclusters and maximal *e*-CCC-Biclusters**. This figure shows: **(Top) **1-CCC-Biclusters obtained from the maximal CCC-Biclusters in Figure 2 by extending them with genes by looking for their approximate patterns in the generalized suffix tree (1-CCC-Biclusters B1_1, B2_1, B3_1, B5_1 and B6_1) or extending them with *e *= 1 contiguous columns at right (1-CCC-Biclusters B1_2, B1_3, B2_2, B4_2, B6_2 and B6_3) or at left (1-CCC-Biclusters B2_3, B3_2, B4_1, B5_2 and B5_3). Note that several of these 1-Biclusters can be defined by more than one expression pattern. This is the case of 1-CCC-Biclusters B2_1, B2_3, B3_2, B4_1 and B4_2, which in fact correspond to maximal 1-CCC-Biclusters (see Figure 5). Other 1-CCC-Biclusters are identified by a single expression pattern. This is the case of 1-CCC-Biclusters B1_1, B1_2, B2_1, B3_1, B5_1, B5_2, B6_1 and B6 2, and also correspond to maximal 1-CCC-Biclusters (see Figure 5). However, the 1-CCC-Biclusters B1_3, B5_3 and B6_3 do not correspond to maximal 1-CCC-Biclusters since they are not row-maximal. **(Bottom) **Maximal 1-CCC-Biclusters B1_3, B5_3 and B6_3 obtained not only by extending maximal CCC-Biclusters B1, B5 and B6 with one contiguous column to the right, left and right, respectively, but also by looking for the patterns in the 1-neighborhood of the patterns *S*_*B*1_3 _= [U U] (columns C1–C2), *S*_*B*5_3 _= [U U] (columns C4–C5) and *S*_*B*6_3 _= [N U] (columns C1–C2). Note however, that even if we replaced the non maximal 1-CCC-Biclusters B1_3, B5_3 and B6_3 (in the top) by the truly maximal 1-CCC-Biclusters (in the bottom) we could only find 16 of the 36 maximal 1-CCC-Biclusters with at least two rows shown in Figure 5 that can be found in the discretized matrix in Figure 1.

Furthermore we cannot obtain *all *maximal *e*-CCC-Biclusters using the set of maximal CCC-Biclusters by: 1) extending them with genes by looking for their approximate patterns in the generalized suffix tree, or 2) extending them with *e *contiguous columns (see Figure 5 and Figure 8). It is also clear from Figure 8 that extending maximal CCC-Biclusters can in fact lead to the discovery of non maximal *e*-CCC-Biclusters. For the reasons stated above we cannot use the same searching strategy used to find maximal CCC-Biclusters when looking for maximal *e*-CCC-Biclusters (*e > *0). We therefore need to explore the relation between finding *e*-CCC-Biclusters and the *Common Motifs Problem*, as explained below.

### Finding *e*-CCC-Biclusters and the common motifs problem

There is an interesting relation between the problem of finding all maximal *e*-CCC-Biclusters, discussed in this work, and the well known problem of finding common motifs (patterns) in a set of sequences (strings). For the first problem, and to our knowledge, no efficient algorithm has been proposed to date. For the latter problem (*Common Motifs Problem*), several efficient algorithms based on string processing techniques have been proposed to date [25,26]. The *Common Motifs Problem *is as follows [26]:

**Common Motifs Problem **Given a set of *N *sequences *S*_*i *_(1 ≤ *i *≤ *N*) and two integers *e *≥ 0 and 2 ≤ *q *≤ *N*, where *e *is the number of errors allowed and *q *is the required quorum, find all models *m *that appear in at least *q *distinct sequences of *S*_*i*_.

During the design of *e*-CCC-Biclustering, we used the ideas proposed in SPELLER [26], an algorithm to find *common motifs *in a set of *N *sequences using a generalized suffix tree *T*. The motifs searched by SPELLER correspond to *words*, over an alphabet Σ, which must occur with at most *e *mismatches in 2 ≤ *q *≤ *N *distinct sequences. Since these words representing the motifs may not be present exactly in the sequences (see SPELLER for details), a motif is seen as an "external" object and called *model*. In order to be considered a *valid model*, a given model *m *of length |*m*| has to verify the *quorum constraint*: *m *must belong to the *e*-neighborhood of a word *w *in at least *q *distinct sequences.

In order to solve the *Common Motifs Problem*, SPELLER builds a generalized suffix tree *T *for the set of sequences *S*_*i *_and then, after some further preprocessing, uses this tree to "spell" the valid models. Valid models verify two properties [26]:

1. All the prefixes of a valid model are also valid models.

2. When *e *= 0, spelling a model leads to one node *v *in *T *such that *L*(*v*) ≥ *q*, where *L*(*v*) denotes the number of leaves in the subtree rooted at *v*.

When *e *> 0, spelling a model leads to a set of nodes *v*_1_, ..., *v*_*k *_in *T *for which , where *L*(*v*_*j*_) denotes the number of leaves in the subtree rooted at *v*_*j*_.

In these settings, and since the occurrences of a model are in fact nodes of the generalized suffix tree *T*, these occurrences are called *node-occurrences *[26]. The goal of SPELLER is thus to identify all valid models by extending them in the generalized suffix tree and to report them together with their set of node-occurrences. We present here an adaptation of the definition of node-occurrence used in SPELLER. In SPELLER, a node-occurrence is defined by a pair (*v*, *v*_*err*_) and not by a triple (*v*, *v*_*err*_, *p*), as in this work. For clarity, SPELLER was originally exemplified [26] in an uncompacted version of the generalized suffix tree, that is, a trie (although it was proposed to work with a generalized suffix tree). However, and as pointed out by the authors, when using a generalized suffix tree, as in our case, we need to know at any given step in the algorithm whether we are at a node or in an edge between nodes *v *and *v'*. We use *p *to provide this information, and redefine node-occurrence as follows:

**Definition 11 (node-occurrence) ***A node-occurrence of a model m is a triple (v, v*_*err*_, *p*), *where v is a node in the generalized suffix tree T and v*_*err *_*is the number of mismatches between m and the string-label of v computed using Hamming(m, string-label(v)). The integer p *≥ 0 *identifies a position/point in T such that:*

*1. If p *= 0: *we are exactly at node v*.

*2. If p *> 0: *we are in E*(*v*), *the edge between father_*v *_and v, in a point p between two symbols in label*(*E*(*v*)) *such that *1 ≤ *p *< |*label*(*E*(*v*))|.

Consider a model *m*, a symbol *α *in the alphabet Σ, a node *v *in *T*, its father *father*_*v*_, the edge between *father*_*v *_and *v*, *E*(*v*), the edge-label of *E*(*v*), *label*(*E*(*v*)) and its edge-length, |*label*(*E*(*v*))|. The modified version of SPELLER described below is based on the following Lemmas (adapted from SPELLER):

**Lemma 5 **(*v*, *v*_*err*_, 0) *is a node-occurrence of a model m' *= *mα, if, and only if:*

*1*. ***Match:***

(*father*_*v*_, *v*_*err*_, 0) *is a node-occurrence of m and label*(*E*(*v*)) = *α*.

The edge-label of *E*(*v*) *has only one symbol and this symbol is α*.

*or*

(*v*, *v*_*err*_, |*label*(*E*(*v*))| -1) *is a node-occurrence of m and label*(*E*(*v*)) [|*label*(*E*(*v*))|] = *α*.

*The last symbol in label*(*E*(*v*)) *is α*.

*2*. ***Substitution:***

(*father*_*v*_, *v*_*err *_-1, 0) *is a node-occurrence of m and label*(*E*(*v*)) = *β *≠ *α*.

*The edge-label of E*(*v*) *has only one symbol and this symbol is not α*.

*or*

(*v*, *v*_*err *_- 1, |*label*(*E*(*v*))| - 1) *is a node-occurrence of m and label*(*E*(*v*)) [|*label*(*E*(*v*))|] = *β *≠ *α*.

*The last symbol in label*(*E*(*v*)) *is not α*.

**Lemma 6 **(*v*, *v*_*err*_, 1) *is a node-occurrence of a model m' *= *mα, if, and only if:*

*1*. ***Match:***

(*father*_*v*_, *v*_*err*_, 0) *is a node-occurrence of m and label*(*E*(*v*))[1] = *α*.

*2*. ***Substitution:***

(*father*_*v*_, *v*_*err *_- 1, 0) *is a node-occurrence of m and label*(*E*(*v*))[1] = *β *≠ *α*.

**Lemma 7 **(*v*, *v*_*err*_, *p*), 2 ≤ *p *< |*label*(*E*(*v*)| *is a node-occurrence of a model m' *= *mα, if, and only if:*

*1*. ***Match:***

(*v*, *v*_*err*_, *p *- 1) *is a node-occurrence of m and label*(*E*(*v*) [*p*] = *α*.

*2*. ***Substitution:***

(*v*, *v*_*err *_- 1, *p *- 1) *is a node-occurrence of m and label*(*E*(*v*)) [*p*] = *β *≠ *α*.

Consider now the discretized matrix *A *obtained from matrix *A' *using the alphabet Σ. We preprocess *A *using the same alphabet transformation used in CCC-Biclustering. Remember that we append the column number to each symbol in the matrix and consider a new alphabet Σ*' *= Σ × {1, ..., |*C*|} (see Figure 3). We will now show that SPELLER can be adapted to extract all right-maximal *e*-CCC-Biclusters from this transformed matrix *A *by building a generalized suffix tree for the set of |*R*| strings *S*_*i *_obtained from each row in *A *and use it to "spell" the valid models using the symbols in the new alphabet Σ*'*.

Given the set of |*R*| strings *S*_*i*_, the number of allowed errors *e *≥ 0 and the quorum constraint 2 ≤ *q *≤ |*R*|, the goal is now to find the set of all *right-maximal *valid models *m*, identifying expression patterns that are present in at least *q *distinct rows *starting and ending at the same columns*. Note that the valid models identified by the original SPELLER algorithm are already row-maximal. However they may be non right-maximal, non left-maximal, and start at different positions in the sequences. Under these settings, the set of node-occurrences of each valid model *m *and the model itself in our modified version of SPELLER identifies one row-maximal, right-maximal *e*-CCC-Bicluster with *q *rows and a maximum of |*C*| contiguous columns. Furthermore, it is possible to find all right-maximal *e*-CCC-Biclusters by fixing the quorum constraint, used to specify the number of rows/genes necessary to identify a model as valid, to the value *q *= 2. In this context, and in order to be able to solve not only Problem 1 but also Problem 2, we adapted SPELLER to consider not only a *row constraint*, 2 ≤ *q*_*r *_≤ |*R*|, but also an additional *column constraint*, 1 ≤ *q*_*c *_≤ |*C*|.

Figure 7 shows the generalized suffix tree used by our modified version of SPELLER when it is applied to the discretized matrix after alphabet transformation in Figure 3. We can also see in this figure the five maximal 1-CCC-Biclusters B1, B2, B3, B4 and B5, already shown in Figure 6, identified by five valid models, when *e *= 1 and the values *q*_*r *_and *q*_*c*_, specifying the row and column constraints, respectively, are set to 3. The maximal 1-CCC-Biclusters B1 to B5 are defined, respectively, by the following valid models: *m *= [D1 U2 D3 U4 N5] (three node-occurrences labeled with B1); *m *= [D2 D3 U4] (three node-occurrences labeled with B2), *m *= [D3 U4 N5] (four node-occurrences labeled with B3), *m *= [N2 D3 U4] (four node-occurrences labeled with B4) and *m *= [U2 D3 U4 D5] (four node-occurrences labeled with B5). It is also possible to observe in this figure that, when *e > *0, *a model can be valid without being right/left-maximal *and that *several valid models may identify the same e-CCC-Bicluster*. For example, *m *= [D1 U2 D3] is valid but it is not right-maximal, *m *= [D3 U4 D5] is also valid but it is not left-maximal, and finally the models *m *= [D1 U2 D3 U4 N5] and *m *= [N1 U2 D3 U4 D5] are both valid but identify the same 1-CCC-Bicluster B1. Figure 4 shows the generalized suffix tree used when *e *= 0, *q*_*r *_= 2 and *q*_*c *_= 1. Since no errors are allowed the generalized suffix tree is the same as the one used by CCC-Biclustering and the maximal 0-CCC-Biclusters identified correspond in fact to the maximal CCC-Biclusters in Figure 2.

In the next section we describe the details of the modified version of SPELLER that we used to identify all right-maximal *e*-CCC-Biclusters. However, and for clarity, we summarize here the main differences between the original version of SPELLER and the modified version (procedure computeRightMaximalBiclusters in the next section), which we use as the first step of the *e*-CCC-Biclustering algorithm. While reading the differences listed below have in mind that in order to be maximal, an *e*-CCC-Bicluster must be row-maximal, right-maximal and left-maximal. Moreover, all the approximate patterns identifying genes in an *e*-CCC-Bicluster must start and end at the same columns.

1. In SPELLER a node-occurrence is defined by a pair (*v*, *v*_*err*_) since (for clarity) the algorithm was exemplified using a trie and not a generalized suffix tree, as explained above. As such we redefined the original concept of node-occurrence to use the triple (*v*, *v*_*err*_, *p*) (see Definition 11), adapted the three original Lemmas in SPELLER to use the new definition of node-occurrence (see Lemma 5, Lemma 6 and Lemma 7), and rewrote SPELLER to use a generalized suffix tree.

2. In SPELLER a model can be valid without being right/left-maximal. As such all models satisfying the quorum constraint are stored for further reporting. This means that the valid models reported by SPELLER are only row-maximal. We only store valid models that cannot be extended to the right without loosing genes, that is valid models which are both row-maximal are right-maximal. This implied modifying the original procedure storeModel in SPELLER in order to include the procedure checkRightMaximality (see procedure spellModels in the next section, for details).

3. In SPELLER the node-occurrences of a valid model can start in any position in the sequences. In our modified version of this algorithm all node-occurrences of a valid model must start in the same position (same column in the discretized matrix) in order to guarantee that they belong to an *e*-CCC-Bicluster. As such we modified the construction of the generalized suffix tree used in SPELLER in order to be constructed using the set of strings corresponding to the set of rows in the discretized matrix after alphabet transformation. We also modified all the procedures used in SPELLER for model extension. Note that it is not possible to modify SPELLER in order to check if a valid model that is right-maximal is also left-maximal. This is so since we can only guarantee that a model is/is not left-maximal once we have computed all valid models corresponding to right-maximal *e*-CCC-Biclusters. This justifies why we need to discard valid models which are not left-maximal in the next step of the algorithm and did not integrate this step in our modified version of SPELLER.

In this context, we also show in the next section that the proposed *e*-CCC-Biclustering algorithm will need *three steps *to identify all maximal *e*-CCC-Biclusters without repetitions: a first step to identify all right-maximal *e*-CCC-Biclusters (for this we use the modified version of SPELLER), a second step to discard all right-maximal *e*-CCC-Biclusters which are not left-maximal, and finally a third step to discard repetitions, that is maximal valid models identifying the same maximal *e*-CCC-Bicluster.

Note that the original SPELLER algorithm does not eliminate repetitions (different valid models with the same set of node-occurrences). Furthermore, we also cannot integrate the elimination of valid models corresponding to the same right-maximal *e*-CCC-Biclusters in our modified version of SPELLER since we need the set of all valid models corresponding to right-maximal *e*-CCC-Biclusters in order to discard valid models which are not left-maximal in the second step of *e*-CCC-Biclustering.

### *e*-CCC-Biclustering: Finding and reporting all maximal *e*-CCC-Biclusters in polynomial time

This section presents *e*-CCC-Biclustering, a polynomial time biclustering algorithm for finding and reporting all maximal CCC-Biclusters with approximate patterns (*e*-CCC-Biclusters), and describes its main steps. Algorithm 1 is designed to solve Problem 2: identify and report all maximal *e*-CCC-Biclusters  such that *I*_*k *_and *J*_*k *_have at least *q*_*r *_rows and *q*_*c *_columns, respectively. The proposed algorithm is easily adapted to solve problem 1 (identify and report all maximal *e*-CCC-Biclusters  without quorum constraints) by fixing the values of *q*_*r *_and *q*_*c *_to the values two and one, respectively. The proposed algorithm is based on the following steps (described in detail below):

**[Step 1] **Computes all valid models corresponding to right-maximal *e*-CCC-Biclusters. Uses the discretized matrix *A *after alphabet transformation, the quorum constraints *q*_*r *_and *q*_*c*_, a generalized suffix tree and a modified version of SPELLER.

**[Step 2] **Deletes all valid models not corresponding to left-maximal *e*-CCC-Biclusters. Uses all valid models computed in Step 1 and a trie.

**[Step 3] **Deletes all valid models representing the same *e*-CCC-Biclusters. Uses all valid models corresponding to maximal *e*-CCC-Biclusters (both left and right) computed in Step 2 and a hash table. Note that this step is only needed when *e > *0.

**[Step 4] **Reports all maximal *e*-CCC-Biclusters.

**Algorithm 1**: *e*-CCC-Biclustering

**Input **: *A*, Σ, *e*, *q*_*r*_, *q*_*c*_

**Output**: Maximal *e*-CCC-Biclusters.

**1 **{*S*_1_, ..., *S*_|*R*|_} ← alphabetTransformation(*A*, Σ)

**2 ***modelsOcc ← *{}

**3 **computeRightMaximalBiclusters(Σ, *e*, *q*_*r*_, *q*_*c*_, {*S*_1_, ..., *S*_|*R*|_}, *modelsOcc*)

**4 **deleteNonLeftMaximalBiclusters(*modelsOcc*)

**5 if ***e *> 0 **then**

**6**   deleteRepeatedBiclusters(*modelsOcc*)

**7 **reportMaximalBiclusters(*modelsOcc*)

Detailed discussions can be found in additional file 2: **algorithmic_complexity_details**.

#### Computing valid models corresponding to right-maximal *e*-CCC-Biclusters

In step 1 of *e*-CCC-Biclustering we compute all valid models *m *together with their node-occurrences *Occ*_*m *_corresponding to right-maximal *e*-CCC-Biclusters. The details are shown in the procedure computeRightMaximalBiclusters below, which corresponds to a modified version of SPELLER.

**Procedure **computeRightMaximalBiclusters

**Input**: Σ, *e*, *q*_*r*_, *q*_*c*_, {*S*_1_, ..., *S*_|*R*|_}, *modelsOcc*

/* The value of *modelsOcc *is updated.         */

**1 ***T*_*right *_← constructGeneralizedSuffixTree({*S*_1_, ..., *S*_|*R*|_})

**2 **addNumberOfLeaves(*T*_*right*_) /* Adds *L*(*v*) to each node *v *in *T*_*right*_.         */

**3 if ***e *≠ 0 **then**

**4**   addColorArray(*T*_*right*_)

   /* Adds *colors*_*v *_to every node *v *in *T*_*right*_: *colors*_*v *_[*i*] = 1, if there is a leaf in the subtree rooted at *v *that is a suffix of*S *_*i*_; *colors*_*v *_[*i*] = 0, otherwise.         */

**5 ***m *← "" /* model *m *is a string [*m *[1] ... *m *[*length*_*m*_-1]]         */

**6 ***length*_*m*_← 0

**7 ***father*_*m*_← "" /* *father*_*m *_is a string [*m*[1] ... *m [length*_*m*_-1]]         */

**8 ** ← 0

**9 ***Occ*_*m *_← {} /* List of node-occurrences (*v, v*_*err*_, *p*)         */

**10 **addNodeOccurrence(*Occ*_*m*_, (root(*T*_*right*_), 0, 0))

**11 ***Ext*_*m *_← {} /* *Ext*_*m *_is the set of possible symbols *α *to extend the model *m*.         */

**12 if ***e *= 0 **then**

**13   forall ***edges E*(*v*_*i*_) *leaving from node *root(*T*_*right*_) *to a node v*_*i *_**do**

**14      if ***label*(*E*(*v*_*i*_))[1] *is not a string terminator ***then**

**15**         addSymbol(*Ext*_*m*_, *label*(*E*(*v*_*i*_))[1])

16 else

**17   forall ***symbols in Σ' ***do**

         /* Σ*' *must be in lexicographic order.         */

**18**      addSymbol(*Ext*_*m*_, *Σ' *[*i*])

**19 ***length*_*m *_← 0

**20 **spellModels(Σ, *e*, *q*_*r*_, *q*_*c*_, *modelsOcc*, *T*_*right*_, *m*, *length*_*m*_, *Occ*_*m*_, *Ext*_*m*_, *father*_*m*_, )

In this procedure we use the transformed matrix *A *as input and store the results in the list *modelsOcc*, which stores triples with the following information (*m*, *genesOcc*_*m*_, *numberOfGenesOcc*_*m*_), where *m *is the model, *genesOcc*_*m *_is a bit vector containing the distinct genes in the node-occurrences of *m*, *Occ*_*m*_, and *numberOfGenesOcc*_*m *_is the number of bits set to 1 in *genesOcc*_*m *_and, therefore, the number of genes where the model occurs. This information is computed using the procedure spellModels described below, which corresponds to a modified version of the procedure with the same name used in SPELLER).

**Procedure **spellModels

/* Called recursively. Stores right-maximal *e*-CCC-Biclusters in *modelsOcc*.         */

**Input **: Σ, *e*, *q*_*r*_, *q*_*c*_, *modelsOcc*, *T*_*right*_, *m*, *length*_*m*_, *Occ*_*m*_, *Ext*_*m*_, *father*_*m*_, 

/* The value of *modelsOcc *is updated.         */

**1 **keepModel(*q*_*r*_, *q*_*c*_, *modelsOcc, T*_*right*_, *m*, *length*_*m*_, *Occ*_*m*_, *father*_*m*_, 

**2 if ***length*_*m *_≤ |*C*| **then**

/* |*C*| is the length of the longest model         */

**3   forall ***symbols α in Ext*_*m *_**do**

**4      if *α ****is not a string terminator ***then**

**5**         maxGenes ← 0/* Sum of *L*(*v*) for all node-occurrences (*v*, *v*_*err*_, *p*) in *Occ*_*mα*_         */

**6**         minGenes← ∞/* Minimum *L*(*v*) in all node-occurrences (*v*, *v*_*err*_, *p*) in *Occ*_*mα*_         */

**7**         *Colors*_*mα *_← {}

**8**         **if ***e *> 0 **then**

**9**            *Colors*_*mα *_[*i*] ← 0, 1 ≤ *i *≤ |*R*|

            /* *colors*_*mα *_[*i*] = 1, if there is a node-occurrence of *m *in *S*_*i*_;         */

            /* *colors*_*mα *_[*i*] = 0, otherwise         */

**10**      *Ext*_*mα *_← {}

**11**      *Occ*_*mα *_← {}

**12**      **forall ***node-occurrences (v, v*_*err*_, *p*) *in Occ*_*m *_**do**

            /* If p = 0 we are at node *v*. Otherwise, we are at edge *E*(*v*) between nodes *father*(*v*) and*v *at point *p* > 0.         */

**13**            **if ***p *= 0 **then**

**14**               extendFromNodeWithoutErrors(Σ, *e*, *T*_*right*_, (*v*, *v*_*err*_, *p*), *m*, *α*, *Occ*_*mα*_, *Colors*_*mα *_, *Ext*_*mα*_, *maxGenes*, *minGenes*)

**15**               **if **(*v*_*err *_<*e*) **then**

**16**                  extendFromNodeWithErrors(Σ, *e*, *T*_*right*_, (*v*, *v*_*err*_, *p*), *m*, *α*, *Occ*_*mα *_, *Colors*_*mα *_, *Ext*_*mα*_, *maxGenes*, *minGenes*)

**17**            **else**

**18**               extendFromEdgeWithoutErrors(*T*_*right*_, Σ, *e*, (*v*, *v*_*err*_, *p*), *m*, *α*, *m*, *Occ*_*mα*_, *Colors*_*mα *_, *Ext*_*mα *_, *maxGenes*, *minGenes*)

**19**               **if ***x*_*err *_<*e ***then**

**20**                  extendFromEdgeWithErrors(Σ, *e*, *T*_*right*_, (*v*, *v*_*err*_, *p*), *m*, *α*, *Occ*_*mα *_, *Colors*_*mα *_, *Ext*_*mα*_, *maxGenes*, *minGenes*)

**21**         **if **modelHasQuorum(*maxGenes, minGenes, Colors*_*mα*_, *q*_*r*_) **then**

**22**            spellModels(Σ, *e*, *q*_*r*_, *q*_*c*_, *modelsOcc*, *T*_*right*_, *mα*, *length*_*m *_+ 1, *Occ*_*mα *_, *Ext*_*mα *_, *father*_*mα*_, *numberOfGenesOcc*_*m*_)

The recursive procedure spellModels (modified to extract valid models corresponding to right-maximal *e*-CCC-Biclusters) is now able to:

1. Use a generalized suffix tree *T*_*right *_and define node-occurrences as triples (*v*, *v*_*err*_, *p*), where *p *is used throughout the algorithm to find out whether we are at node *v *(*p *= 0) or in an edge *E*(*v*) between nodes *v *and *father*_*v *_(*p > *0).

2. Check if a valid model *m *corresponds to a right-maximal *e*-CCC-Bicluster. This is performed using the procedure checkRightMaximality inside the procedure keepModel. This procedure deletes from the list of stored models, *modelsOcc*, a valid model *m *when the result of its extension with a symbol *α*, *mα*, is also a valid model and the set of node-occurrences of *mα*, *Occ*_*mα*_, has as many genes as the set of node-occurrences of its father *m*, *Occ*_*m*_. When this is the case, *m *no longer corresponds to a right-maximal *e*-CCC-Bicluster since its expression pattern can be extended to the right with the symbol *α *without losing genes.

3. Restrict the extensions of a given model *m*, *Ext*_*m*_, to the level of the model in the generalized suffix tree (column of the last symbol in *m*). When we are extending a model *m *with a symbol *α *(eventually extracting a valid model *mα*), the column number of the last symbol in *m*, *m *[*length*_*m*_], is *C*(*m *[*length*_*m*_]), where *C*(*m *[*length*_*m*_]) ∈ {1, ..., |*C*|}, and errors are still allowed, *α *can only be one of the symbols in the set , where  corresponds to the subset of elements in Σ*' *whose column is equal to *C*(*m *[*length*_*m*_])) + 1. For example, if Σ = {*D*, *N*, *U*} and the model *m *= [D1] is being extended, the possible symbols *α *with which *m *can be extended to *mα *must be in  = {*D*2. *N *2, *U *2}. In the same way, if *m *= [D2 U3], the possible symbols *α *with which *m *can be extended to *mα *are in  = {*D*4, *N *4, *U *4}.

The algorithmic details of the procedures and functions called in the recursive procedure spellModels are described in additional file 2: **algorithmic_complexity_details**.

#### Deleting valid models not corresponding to left-maximal *e*-CCC-Biclusters

In step 2 of *e*-CCC-Biclustering (details in procedure deleteNonLeftMaximalBiclusters below), we remove from the valid models stored in *modelsOcc *(identifying right-maximal *e*-CCC-Biclusters) those not corresponding to left-maximal *e*-CCC-Biclusters. These models are removed from *modelsOcc *by first building a trie with the reverse patterns of all (right-maximal) models *m *and storing the number of genes in *numberOfGenesOcc*_*m *_in its corresponding node in the trie. After this, it is sufficient to mark as "non left-maximal" any node in the trie that has at least one child with as many genes as itself. This is easily achieved by performing a depth-first search (*dfs*) of the trie and computing, for each node, the maximum value amongst the values of *numberOfGenesOcc*_*m *_stored in its children. The models whose corresponding node in the trie is marked as "non left-maximal" are then removed from *modelsOcc*.

**Procedure **deleteNonLeftMaximalBiclusters

**Input**: *modelsOcc*

/* The value of *modelsOcc *is updated. */

**1 ***T*_*left*_← createTrie ()

/* Array which will store references to nodes in *T*_left _*/

**2 ***R*_*nodes*_← {}

**3 foreach ***model and occurrences (m, genesOcc*_*m*_, *numberOfGenesOcc*_*m*_) *in modelsOcc ***do**

**4**   *m*_*r*_← ReverseModel(*m*)

**5**   *nodeRepresentingModel ← *addReverseModelToTrie(*T*_*left*_, *m*_*r*_)

      /* Each node in *T*_*left *_stores two integers: 1) the number of genes in the model it represents, *genes*_*v *_(0 if it does not represent the end of a model); and 2) the maximum number of genes in the subtree rooted at*v*, (computed later). Both these values are initialized with 0.            */

**6**   addNumberOfGenes(*nodeRepresentingModel,numberOfGenesOcc*_*m*_)

**7**   addReferenceToNode(*R*_*nodes*_, *nodeRepresentingModel*)

**8 forall ***nodes v in T*_*left *_**do**

      /* Performed using a depth-first search (dfs)            */

**9**   **if ***genes*_*v *_> 0 **then**

         /* Node *v *represents a model and is potentially left-maximal.            */

**10**      Mark *v *as "left-maximal"

**11**   **else**

**12**      Mark *v *as "non left-maximal"

**13**   Compute the maximum number of genes in the subtree rooted at *v*

**14 foreach ***node v in T*_*left *_**do**

   /* Performed using a depth-first search (dfs)            */

**15**   **if ***genes*_*v *_> 0 *and genes*_*v *_= **then**

**16**      Mark *v *as "non left-maximal"

**17 ***p*_*modelsOcc *_← 0

**18 foreach ***model and occurrences (m, genesOcc*_*m*_, *numberOfGenesOcc*_*m*_) *in modelsOcc ***do**

**19**   **if ***R*_*nodes *_[*p*_*modelsOcc*_] *is marked as *"*non-left maximal" ***then**

**20**      deleteModelAndOccurrences(*modelsOcc, m*)

**21**   *p*_*modelsOcc *_← *p*_*modelsOcc *_+ 1

#### Deleting valid models representing the same *e*-CCC-Biclusters

When errors are allowed, different valid models may identify the same *e*-CCC-Bicluster. Step 3 of *e*-CCC-Biclustering, described in detail in procedure deleteRepeatedBiclusters below, uses a hash table to remove from *modelsOcc *all the valid models that, although maximal (left and right), identify repeated *e*-CCC-Biclusters. This is needed because all valid models *m *with the same first and last columns and the same set of genes represent the same maximal *e*-CCC-Bicluster.

**Procedure **deleteRepeatedBiclusters

**Input**: *modelsOcc*

/* The value of *modelsOcc *is updated.         */

**1 ***H ← *createHashTable()

**2 foreach ***model and occurrences (m, genesOcc*_*m*_, *numberOfGenesOcc*_*m*_) *in modelsOcc ***do**

**3**   *firstColumn*_*m *_= *C*(*m* [1])

**4**   *lastColumn*_*m *_= *C*(*m *[*length*_*m*_])

**5**   *key *← createKey(*firstColumn, lastColumn, genesOcc*_*m*_)

**6**   *value *← (*firstColumn*, *lastColumn*, *genesOcc*_*m*_)

**7**   **if **containsKey(*H*, *key*) **then**

**8**      *value*_*key *_← getValue(*H, key*)

**9**      **if ***value *= *value*_*key *_**then**

         /* *H *already has a value representing the same *e*-CCC-Bicluster         */

**10**         deleteModelAndOccurrences(*modelsOcc, m*)

**11**      **else**

**12**         insertKeyValue(*key, value*)

**13**   **else**

**14**      insertKeyValue(*key*, *value*)

#### Reporting all maximal *e*-CCC-Biclusters

After the three main steps of *e*-CCC-Biclustering the list *modelsOcc *stores all valid models corresponding to maximal *e*-CCC-Biclusters satisfying the quorum constraints *q*_*r *_and *q*_*c*_. In this context, the reporting procedure reportMaximalBiclusters, described below, lists these *e*-CCC-Biclusters using the information stored in the model *m *(needed to identify the expression pattern and the columns in each *e*-CCC-Bicluster) and the bit vector *genesOcc *(needed to identify the genes in the *e*-CCC-Bicluster).

**Procedure **reportMaximalBiclusters

**Input**: *modelsOcc*

**1 foreach ***model and occurrences (m, genesOcc*_*m*_, *numberOfGenesOcc*_*m*_) *in modelsOcc ***do**

**2**   *firstColumn*_*m *_= *C*(*m* [1])

**3**   *lastColumn*_*m *_= *C*(*m *[*length*_*m*_])

**4**   print(*m*, *firstColumn*_*m*_, *lastColumn*_*m*_, *genesOcc*_*m*_)

### *e*-CCC-Biclustering: Complexity analysis

In this section we sketch an analysis of the complexity of *e*-CCC-Biclustering. For a detailed complexity analysis see additional file 2: **algorithmic_complexity_details**.

Given a discretized matrix *A *with |*R*| rows and |*C*| columns, the alphabet transformation performed using the procedure alphabetTransformation takes *O*(|*R*||*C*|) time.

The complexity of computing all valid models corresponding to right-maximal *e*-CCC-Biclusters using procedure computeRightMaximalBiclusters takes *O*(|*R*|^2^|*C*|^1 + *e*^|Σ|^*e*^) operations. The construction of *T*_*right *_and the computation of *L*(*v*) for all its nodes takes *O*(|*R*||*C*|) time each, using Ukkonen's algorithm with appropriate data structures, and a *dfs*, respectively. The increase in the alphabet size from |Σ| to |*C*||Σ| due to the alphabet transformation does not affect the *O*(|*R*||*C*|) construction and manipulation of the generalized suffix tree [9]. When *e *> 0, adding the color array to all nodes in *T*_*right *_takes *O*(|*R*|^2^|*C*|) time. Initializing *Ext*_*m *_takes *O*(|*C*||Σ|) and spellModels is *O*(|*R*|^2^|*C*|^1 + *e*^|Σ|^*e*^). The complexity of this step of the algorithm is bounded by the complexity of spellModels and is thus *O*(|*R*|^2^|*C*|^1+*e*^|Σ|^*e*^). The complexity of deleting from *modelsOcc *all valid models that are not left-maximal using procedure deleteNonLeftMaximalBiclusters is *O*(|*R*||*C*|^2+*e*^|Σ|^*e*^). Since the number of models in *modelsOcc *is *O*(|*R*||*C*|^1+*e*^|Σ|^*e*^) and the size of the models is *O*(|*C*|), the trie *T*_*left *_can be constructed and manipulated in *O*(|*R*||*C*|^2 + *e*^|Σ|^*e*^).

The complexity of deleting from *modelsOcc *all models representing the same *e*-CCC-Biclusters with procedure deleteRepeatedBiclusters takes *O*(|*R*|^2^|*C*|^1 + *e*^|Σ|^*e*^). Since computing the hash key for each of the *O*(|*R*||*C*|^1 + *e*^|Σ|^*e*^) models in *modelsOcc *takes *O*(|*R*|) time, the overall complexity of this step is *O*(|*R*|^2^|*C*|^1 + *e*^|Σ|^*e*^).

Since the number of genes in *genesOcc*_*m *_is *O*(|*R*|) and computing the first and last column of the valid model *m *takes constant time, reporting all maximal *e*-CCC-Biclusters using procedure reportMaximalBiclusters is *O*(|*R*|^2^|*C*|^1+*e*^|Σ|^*e*^).

Therefore, the asymptotic complexity of the proposed *e*-CCC-Biclustering algorithm is *O*(max (|*R*|^2^|*C*|^1+*e*^|Σ|^*e*^, |*R*||*C*|^2 + *e*^|Σ|^*e*^)). However, in most cases of interest |*R*| >>|*C*| and the complexity becomes *O*(|*R*|^2^|*C*|^1+*e*^|Σ|^*e*^). Moreover, when *e *= 0, CCC-Biclustering [9,22] can be used to obtain *O*(|*R*||*C*|).

### Extensions to handle missing values, anticorrelated and scaled expression patterns

**Figure 9:**
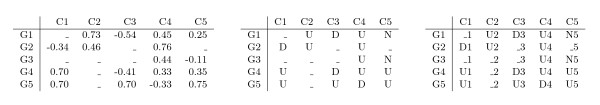
**Illustrative example with missing values**. This figure shows: **(Left) **Original expression matrix, **(Middle) **Discretized matrix and **(Right) **Discretized matrix after alphabet transformation.

In this section we present extensions to *e*-CCC-Biclustering able to handle missing values and discover anticorrelated (opposite patterns) and scaled (patterns with different expression rates) expression patterns. In the subsections below we consider the illustrative example in Figure 9, corresponding to a modified version of the example in Figure 1. We now assume that some expression values are missing.

#### Handling missing values

Since *e*-CCC-Biclustering cannot deal with missing values directly, genes with missing values have to be removed, or missing values have to be filled, as a preprocessing step. In this section we present extensions that enable direct processing of the expression matrix with missing values. Our goal is to consider all available time points and thus always include the expression pattern of a gene as input to the extended version of the algorithm. Nevertheless genes with more than a predefined percentage of missing values can still be discarded in a preprocessing step.

Dealing with missing values in *e*-CCC-Biclustering is straightforward and can be performed in two ways:

1. Considering missing values as valid errors.

2. "Jumping over" missing values.

In order to consider missing values as valid errors we modify *e*-CCC-Biclustering as follows:

• The initialization of *Ext*_*m *_in procedure computeRightMaximalBiclusters must include the symbol used for missing value, when *e *> 0, and ignore all edges descending from the root starting with this symbol, when *e *= 0.

• The extension of a model *m *with a symbol *α *in spellModels must take into account the following: *α *can either be, or not be, the symbol used for missing value, depending on whether we are performing an *extension without errors *or performing an *extension with errors*, respectively.

For details, see procedures extendFromNodeWithoutErrors and extendFromEdgeWithoutErrors, in case of extensions without errors, or procedures extendFromNodeWithErrors and extendFromEdgeWithErrors, in case of extensions with errors. These procedures are called in spellModels and described in additional file 2: **algorithmic_complexity_details**.

Consider the illustrative example in Figure 9, where some gene expression values are missing.

**Figure 10:**
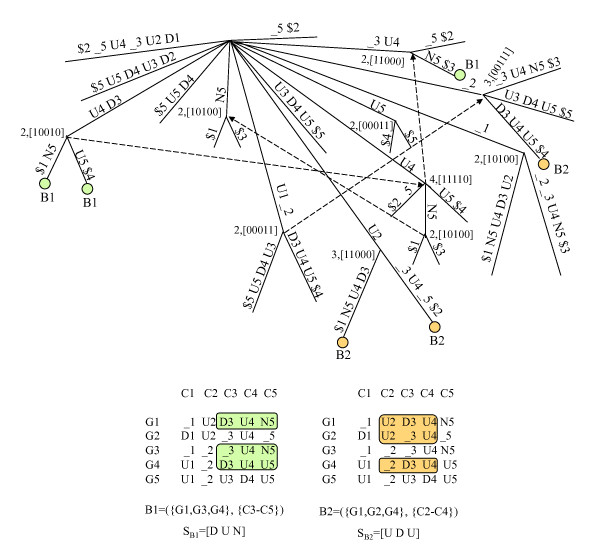
***e*-CCC-Biclusters extended to consider missing values as valid errors**. This figure shows: **(Top) **Generalized suffix tree used by *e*-CCC-Biclustering extended to consider missing values as valid errors when applied to the transformed matrix in Figure 9. The circles labeled with B1 and B2 identify the node-occurrences of the two maximal 1-CCC-Biclusters discovered when *e *= 1 and *q*_*e *_= *q*_*c *_= 3; **(Bottom) **Maximal 1-CCC-Biclusters corresponding, respectively, to the valid models *m *= [D3 U4 N5] (three node-occurrences labeled with B1) and *m *= [U2 D3 U4] (three node-occurrences labeled with B2).

Figure 10 shows the generalized suffix tree *T*_*right *_and the two maximal 1-CCC-Biclusters (B1 and B2) identified by two valid models when *e *= 1, *q*_*r *_= *q*_*c *_= 3 and missing values are considered as valid errors.

In order to "jump over" missing values we modify *e*-CCC-Biclustering as follows:

• After alphabet transformation, we construct the generalized suffix tree *T*_*right*_, used in procedure computeRightMaximalBiclusters, using the set of strings without missing values , where *r*_*i *_is the number of contiguous sets of symbols without missing values in row *i*. The set of substrings of each string *S*_*i *_(gene *i*), , is inserted in *T *using the *same *terminator $*i*.

Consider, for example, the string corresponding to the expression pattern of gene G2 in the illustrative example in Figure 9. In this case, and in order to "jump over" the missing value in the time points C3 and C5, we insert in *T*_*right *_two strings corresponding to each of the two contiguous sets of symbols without missing values in the expression pattern of G2:  = [D1 U2 $2] and  = [U4 $2]. Note that the same terminator $2 is used for all the substrings of row *i*:  and .

**Figure 11:**
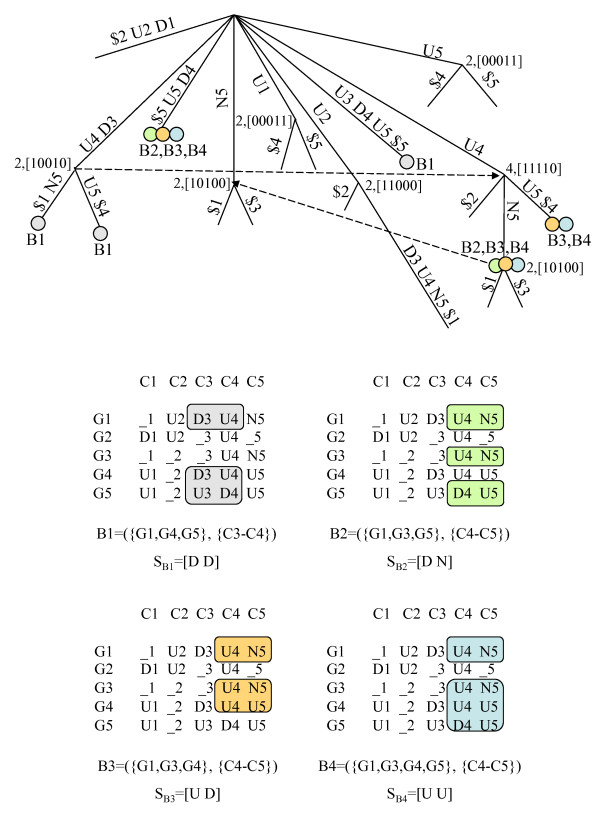
***e*-CCC-Biclusters extended to "jump over" missing values**. This figure shows: **(Top**) Generalized suffix tree used by *e*-CCC-Biclustering extended to "jump over" missing values when applied to the transformed matrix in Figure 9. The circles labeled with B1, B2, B3 and B4 identify the node-occurrences of the four maximal 1-CCC-Biclusters discovered when *e *= 1, *q*_*e *_= 3 and *q*_*c *_= 2; **(Bottom) **Maximal 1-CCC-Biclusters corresponding, respectively, to the valid models *m *= [D2 D3] (three node-occurrences labeled with B1), *m *= [D4 N5] (three node-occurrences labeled with B2), *m *= [U4 D5] (three node-occurrences labeled with B3) and *m *= [U4 U5] (three node-occurrences labeled with B4).

Figure 11 shows the generalized suffix tree *T*_*right *_constructed for the matrix after alphabet transformation in Figure 9 together with the four maximal 1-CCC-Biclusters (B1, B2, B3 and B4), identified by four valid models, when *e *= 1, *q*_*r *_= 3, *q*_*c *_= 2 and the algorithm "jumps over" missing values.

The asymptotic complexity of both versions of this extended version of *e*-CCC-Biclustering remains *O*(max (|*R*|^2^|*C*|^1+*e*^|Σ|^*e*^, |*R*||*C*|^2+*e*^|Σ|^*e*^)). When *e *= 0, a modified version of CCC-Biclustering [27] can be used to achieve the linear time complexity *O*(|*R*||*C*|), if repeated CCC-Biclusters are not filtered. In order to eliminate repetitions, the asymptotic complexity is now *O*(|*R*|^2^|*C*|).

#### Handling anticorrelated expression patterns

Given the importance of anticorrelation relationships in the study of transcription regulation using time series expression data we present here the extension of *e*-CCC-Biclustering to extract maximal *e*-CCC-Biclusters with sign-changes, that is maximal *e*-CCC-Biclusters allowing genes with opposite expression patterns. We first define formally the concepts of opposite expression pattern, *e*-CCC-Bicluster with sign-changes, and maximal *e*-CCC-Bicluster with sign-changes:

**Definition 12 (***e***-CCC-Bicluster with Sign-Changes) ***An e-CCC-Bicluster with sign-changes A*_*IJ *_*is an e-CCC-Bicluster where all the strings S*_*i *_*that define the expression pattern of each of the genes in I are either in the e-Neighborhood of the expression pattern S that defines the e-CCC-Bicluster, or in the e-neighborhood of its opposite expression pattern S*^-1^*: S*_*i *_∈ *N *(*e; S*) *or S*_*i *_∈ *N *(*e*, *S*^-1^), ∀*i *∈ *I*.

**Definition 13 (Maximal ***e***-CCC-Bicluster with Sign-Changes) ***An e-CCC-Bicluster with sign-changes A*_*IJ *_*is maximal if it is row-maximal, left-maximal and right-maximal. This means that no more rows or ****contiguous ****columns can be added to I or J, respectively, maintaining the coherence property in Definition 12*.

In order to discover maximal *e*-CCC-Biclusters with sign-changes we modify *e*-CCC-Biclustering as follows:

• We construct the generalized suffix tree *T*_*right*_, used in procedure computeRight MaximalBiclusters, for the set of strings *S*_*i *_∈ {*S*_1_, ..., *S*_|*R*|_} obtained after alphabet transformation and insert in *T*_*right *_the set of opposite patterns of these strings . Since we use string terminators {$1, ..., $|*R*|} for the expression patterns *S*_*i *_and {$(|*R*| + 1,..., $(2|*R*|)} for their opposite patterns  it is easy to compute the color arrays in *T*_*right *_in *O*(|*R*|) time and space. Notet hat we still use a color array with a maximum of |*R*| bits and not 2|*R*| bits.

• When the extension to "jump over" missing values is considered, we construct *T*_*right *_for the set of strings  and their opposite patterns .

**Figure 12:**
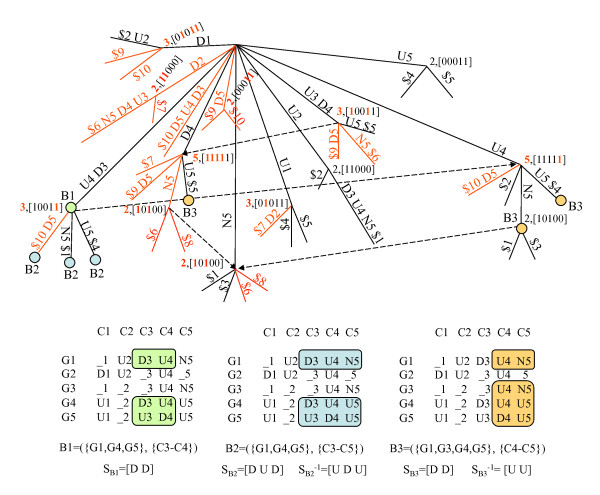
***e*-CCC-Biclusters extended to "jump over" missing values and allow anticorrelation**. This figure shows: **(Top) **Generalized suffix tree used by *e*-CCC-Biclustering extended to "jump over" missing values and extract *e*-CCC-Biclusters with sign-changes when applied to the transformed matrix in Figure 9. The circles labeled with B1, B2 and B3 identify the node-occurrences of the three maximal 1-CCC-Biclusters discovered when *e *= 1, *q*_*e *_= 3 and *q*_*c *_= 2; **(Bottom) **Maximal 1-CCC-Biclusters corresponding, respectively, to the valid models *m *= [D3 D4] (B1), *m *= [D3 U4 D5] and *m*^-1 ^= [U3 D4 U5] (B2), and *m *= [U4 U5] and *m*^-1 ^= [D4 D5] (B3).

Figure 12 shows the generalized suffix tree *T*_*right *_and the three maximal 1-CCC-Biclusters (B1, B2 and B3), identified by three valid models, when *e *= 1, *q*_*r *_= 3 and *q*_*c *_= 2. In this example, the extension "jump over" missing values was used to handle missing values.

The asymptotic complexity of this extended version of *e*-CCC-Biclustering remains *O*(max (|*R*|^2^|*C*|^1+*e*^|Σ|^*e*^, |*R*||*C*|^2+*e*^|Σ|^*e*^)). Note however that, although the asymptotic complexity does not change the constant of proportionality is higher. When *e *= 0, a modified version of CCC-Biclustering [27] can again be used to achieve the linear time complexity *O*(|*R*||*C*|), if repeated CCC-Biclusters are not filtered. However, removing repeated CCC-Biclusters takes *O*(|*R*|^2^|*C*|).

#### Handling scaled expression patterns

Since different genes can have different expression rates, we propose *e*-CCC-Biclustering with scaled expression patterns. These extensions allow the shifting of gene expression patterns up to *K *symbols up and down, in order to potentially find maximal *e*-CCC-Biclusters that would not be found due to different gene expression rates. The value of *K *is an integer between 1 and (|Σ| - 1), where Σ is the set of symbols used to discretize the original expression matrix, in lexicographic order.

In the general case, and in order to shift the expression pattern of the genes *K *symbols up and down we consider a pair of *K *symbol alphabets: Σ^↑ ^and Σ^↓^. These alphabets make it possible to shift all the symbols in |Σ| the desired *K *symbols up and down. Assuming the three alphabets Σ, Σ^↑ ^and Σ^↓ ^are in lexicographic order and thus their symbols respect the ordering Σ^↓ ^[1] < ... < Σ^↓ ^[*K*] < Σ [1] < ... < Σ [|Σ|] <Σ^↑^[1] < ... < Σ^↑ ^[*K*], the alphabet  = Σ^↓ ^∪ Σ ∪ Σ^↑ ^is also in lexicographic order.

For illustration purposes, consider Σ = {*D*, *N*, *U*}, *K *= (|Σ| - 1) = 2, and the illustrative example in Figure 9. In this case, and in order to shift the expression pattern of the genes *K *= 2 symbols up and down, we need to consider, for example, the *K *= 2 symbol alphabets Σ^↑ ^= {*V*, *W*} and Σ^↓ ^= {*B*, *C*}. The three symbols in Σ are then shifted *K *= 2 symbols up and down using the following three pairs of alphabets:  = {*N*, *U*} and  = {*B*, *C*};  = {*U*, *V*} and  = {*C*, *D*}; and  = {*V*, *W*} and  = {*D*, *N*}. Thus,  = {*B*, *C*, *D*, *N*, *U*, *V*, *W*}, in this specific case.

We define *e*-CCC-Bicluster with scaled patterns and the notion of maximality as follows:

**Definition 14 (***e***-CCC-Bicluster with Scaled Patterns) ***An e-CCC-Bicluster with scaled patterns*

*A*_*IJ *_*is an e-CCC-Bicluster where all the strings S_*i *_that define the expression pattern of each of the genes in I are either in the e-Neighborhood of the expression pattern S, that defines the e-CCC-Bicluster, or in the e-neighborhood of the patterns resulting from shifting its expression pattern S K symbols up*, , *or K symbols down*, , *where K is an integer and K *∈ [1, ..., |Σ| - 1]. *This means S*_*i *_∈ <*N *(*e*, *S*) ∨ *S*_*i *_∈ *N *(*e*, *S*^↑^) ∨ *S*_*i *_∈ *N *(*e*, *S*^↓^), ∀*i *∈ *I*.

**Definition 15 (Maximal ***e***-CCC-Bicluster with Scaled Patterns) ***An e-CCC-Bicluster with scaled patterns A*_*IJ *_*is maximal if it is row-maximal, left-maximal and right-maximal. This means that no more rows can be added to the set of rows I and no ****contiguous ****columns can be added to the set of columns J while maintaining the coherence property in Definition 14*.

In order to discover *e*-CCC-Biclusters with scaled patterns we modify *e*-CCC-Biclustering as follows:

• We construct the generalized suffix tree *T*_*right*_, used in procedure computeRight MaximalBiclusters, for the set of strings *S*_*i *_= {*S*_1_, ..., *S*_|*R*|_} and insert in *T*_*right *_the patterns resulting from shifting the expression pattern *S*_*i *_*K *symbols up and down.

Since we use string terminators $1, ..., $|*R*| for the expression patterns *S*_*i *_and $(|*R*| + 1), ..., $(|*R*| + 2 × *K *× |*R*|) for shifted patterns it is easy to compute the colors arrays in *T*_*right *_in *O*(|*R*|) time and space.

• When the extension to "jump over" missing values is considered, we construct *T*_*right *_for the set of strings  together with their corresponding set of shifted patterns *K *symbols up and down.

The asymptotic complexity of *e*-CCC-Biclustering with scaled patterns is *O*(*K*|*R*|^2^|*C*|^1+*e*^|Σ|^*e*^). When *e *= 0, a modified version of CCC-Biclustering [27] can be used to obtain *O*(*K*|*R*||*C*|), or *O*(*K*|*R*|^2^|*C*|) if repetitions are discarded.

### Alternative ways to compute approximate expression patterns

In this section we describe alternative ways to compute the errors allowed in the approximate patterns, which can reveal to be more suitable depending on the specific problem under study. The proposed *e*-CCC-Biclustering algorithm can be modified in order to cope with the three different kinds of errors described below: *restricted errors*, *alphabet range weighted errors*, and *pattern length adaptive errors*.

#### Restricted errors

The *e*-CCC-Biclustering algorithm allows *general errors*, that is, substitutions of the symbols *A*_*ij *_in the *e*-CCC-Bicluster *A*_*IJ *_by any symbol in the alphabet  but *A*_*ij*_. Considering approximate expression patterns having this kind of errors is specially relevant to minimize the negative effect of *measurement errors*, generally occurring during the microarray experiments, in the ability of the algorithm to identify relevant expression patterns. However, if we are specially interested in minimizing the also problematic effects of potential *discretization errors*, introduced due to poor choice of discretization thresholds or number of symbols, we can consider *restricted errors*, that is, substitutions of the symbols *A*_*ij *_by the lexicographically closer symbols (*neighbors*) in .

In general, when restricted errors are considered, the allowed substitutions for any symbol *A*_*ij *_are in the set , where  is the position of *A*_*ij *_in  and *z *is a value in  that specifies the number of neighbors both to the left and to the right of  that are considered valid errors. Note that this set with the allowed symbols to substitute the symbol in *A*_*ij *_has a maximum of (2*z*) elements. Furthermore, the exact number of elements depends both on the number of considered neighbors, *z*, and on the position of *A*_*ij *_in the alphabet , *p*. If  then the errors are not restricted. For example, when general errors are allowed, Σ = {*D*, *N*, *U*}, and *m *= [U2 D3 U4 D5], D5 can be substituted by N5 and U5 in  = {*D*5, *N*5, *U*5} leading to the 1-CCC-Bicluster B5 = ({G1, G2, G4},{C2–C5}) in Figure 7. However, if only restricted errors with *z *= 1 are allowed, D5 can only be substituted by {*N*5} leading to 1-CCC-Bicluster B = ({G1, G2},{C2–C5}).

#### Alphabet range weighted errors

When the alphabet Σ used to discretize the data has many symbols, we can either restrict the errors allowed in the approximate patterns to a neighborhood around the symbol, or to consider alphabet range weighted errors. In the last case, we weight the errors according to the percentage of the total alphabet range they correspond to. For example, if Σ has 10 symbols, an error consisting of a substitution between symbols Σ[1] and Σ[3] should get a weight of 2/9 ~ 0.22 and not a weight of 1 (as happens to all errors in the definition of *e*-CCC-Bicluster). This means that in general an error from symbol Σ[*i*] to symbol Σ [*j*], considering that Σ is in lexicographic order and *i *<*j*, is weighted as , where . Since |Σ| - 1 is the maximum amplitude error, , when *i *= 1 and *j *= |Σ|. Furthermore, , each time *i *= *j *and no error occurred. In these settings, a node-occurrence can be extended with errors if the *weighted sum of the errors *already found is less than *e*.

#### Pattern length adaptive errors

The definition of an *e*-CCC-Bicluster *A*_*IJ *_states that the expression pattern *S*_*i *_of each gene in *I *must be in the *e*-Neighborhood of an expression pattern *S *that defines the *e*-CCC-Bicluster. This implies that the maximum number of errors *e *is fixed, and, as such, it does not take into account the length of the expression pattern  of each individual *e*-CCC-Bicluster *B*_*k*_. Since allowing *e *errors in an expression pattern of a few columns is not the same as allowing *e *errors in longer expression patterns, we propose the use of *pattern length adaptive errors *(the longer the pattern the more errors can be considered during the model extension process). In this context, we believe that a valuable extension to *e*-CCC-Biclustering is to allow not a fixed number of errors *e *per gene in , but an adaptive number of errors, per gene and per *e*-CCC-Bicluster *B*_*k*_, which is computed using a percentage of the number of columns in  . As such the goal is to modify the algorithm in order to find and report all maximal *δ*-CCC-Biclusters (defined below) instead of all maximal *e*-CCC-Biclusters using Lemma 8 and Lemma 9 (described below).

**Definition 16 (*δ*-CCC-Bicluster) ***A contiguous column coherent bicluster A*_*IJ *_*with pattern length adaptive errors, δ-CCC-Bicluster, is a CCC-Bicluster where all the strings S*_*i *_*that define the expression pattern of each of the genes in I are in the δ *-*Neighborhood of an expression pattern S that defines the δ-CCC-Bicluster: S*_*i *_∈ *N *(*δ*, *S*), ∀*i *∈ *I. The value of δ is computed using a percentage of the number of columns in J: δ *= *α*|*J*|, *where α *∈ [0, ..., 1].

**Lemma 8 ***A δ-CCC-Bicluster A*_*IJ *_*is an e-CCC-Bicluster with e *= *δ *= *α*|*J*| *errors per gene in I*.

**Lemma 9 ***The δ-Neighborhood of a string S, N*(*δ*, *S*), *where δ *= *α*|*J*|, *α *∈ [0, ..., 1] *and *|*J*| ∈ [1, ..., |*C*|], *contains **elements, where ϵ *= *α*|*C*|.

### Scoring *e*-CCC-Biclusters using statistical significance and similarity measures

Since applying biclustering to real gene expression matrices can produce hundreds or even thousands of biclusters, an objective evaluation of the quality of the biclusters discovered is crucial. In fact, the inspection of biclustering results can be prohibitive without an efficient scoring approach which enables sorting and filtering the results according to a statistical scoring criterion. The statistical significance of the results can then be combined with measures of biological significance in order to produce a set of interesting and potentially useful biclusters, both from the statistical and biological point of view. For *e*-CCC-Biclusters, we propose the use of a scoring criterion, which combines two criteria:

1. Statistical significance of expression patterns.

2. Similarity measure between overlapping *e*-CCC-Biclusters.

In this work, we extend the concept of statistical significance of perfect expression patterns, proposed for CCC-Biclusters [9], in order to compute the statistical significance of approximate expression patterns. Based on this scoring criterion, the *p*-value of each *e*-CCC-Bicluster is computed and those not passing a Bonferroni corrected statistical significance test at a predefined level are discarded. Biclusters are then sorted by increasing order of their *p*-value and, when several of them overlap more than a predefined threshold, only the most significant are kept.

Note that although we use the stringent Bonferroni correction for multiple testing at the 1% level to guarantee that only the highly significant *e*-CCC-Biclusters are considered for further analysis, other less conservative statistical corrections can be used, thus considering more *e*-CCC-Biclusters as highly significant. Other significance levels can also be used. Moreover, even though we also use a stringent threshold in the overlapping filter (only *e*-CCC-Biclusters overlapping less than 25% are further analyzed), other overlapping thresholds can be used, thus allowing the analysis of a larger number of *e*-CCC-Biclusters, although potentially increasing the number of redundant biclusters.

In what follows we describe how to compute the statistical significance of an *e*-CCC-Bicluster using its expression pattern and describe the similarity score used to compare *e*-CCC-Biclusters.

#### Statistical significance

We proposed to measure the statistical significance of an *e*-CCC-Bicluster *B *of size |*I*| × |*J*|, where *I *is the set of genes and *J *is the set of contiguous time-points, and expression pattern *p*_*B*_, against the null hypothesis, *H*_0_, that assumes that the expression values of genes evolve independently.

Under the null hypothesis, it is possible to compute, using reasonable simplifying assumptions, the probability of an *e*-CCC-Bicluster of the considered size and expression pattern occurring by chance in an expression matrix with |*R*| genes and |*C*| time points. The value of this probability is obtained by computing the *tail of the binomial distribution*, which gives the probability of an event with probability *p *occurring *k *or more times in *n *independent trials: .

The statistical significance of an *e*-CCC-Bicluster *B *is thus the value of *p*-value(*B*), which is computed by obtaining the probability of a random occurrence under *H*_0 _of the expression patterns in the *e*-Neighborhood of the expression pattern *p*_*B *_defining the *e*-CCC-Bicluster, *N *(*e, p*_*B*_), *k *= |*I*| - 1 times in *n *= |*R*| - 1 independent trials, where *I *is the number of genes in *B *and |*R*| is the total number of genes in the gene expression matrix. This is performed using the simplifying assumption that the probability of occurrence of a specific expression pattern in the *e*-Neighborhood of the pattern *p*_*B*_, *N *(*e*, *p*_*B*_), is adequately modeled by a first order Markov Chain, with state transition probabilities obtained from the values in the corresponding columns in the matrix. In the general case,



where |*N*(*e*, *p*_*B*_)| and *N *(*e*, *p*_*B*_) [*i*] are, respectively, the number of patterns and the *i*th pattern in the *e*-Neighborhood of the pattern *p*_*B*_. As an example, consider the computation of *P *(*N *(*e*, *p*_*B*_)) when *B *is the *e*-CCC-Bicluster B1 = ({*G*1, *G*2, *G*4}, {*C*1 - *C*4}) in Figure 7 with *p*_*B *_= [D1 U2 D3 U4]. Since, in this case, *e*-CCC-Biclustering was applied using *e *= 1, we have to compute *P*(*N*(1, [D1 U2 D3 U4])), which has, in this case, the following set of elements: {[D1 U2 D3 U4], [N1 U2 D3 U4], [U1 U2 D3 U4], [D1 D2 D3 U4], [D1 N2 D3 U4], [D1 U2 N3 U4], [D1 U2 U3 U4], [D1 U2 D3 D4], [D1 U2 D3 N4]}.

In this context, the value of *P*(*N*(1, [D1 U2 D3 U4])) is computed as follows: *P*(*N*(1, [D1 U2 D3 U4])) = *P*([D1 U2 D3 U4]) + *P*([N1 U2 D3 U4]) + *P*([U1 U2 D3 U4]) + *P*([D1 D2 D3 U4]) + *P*([D1 N2 D3 U4]) + *P*([D1 U2 N3 U4]) + *P*([D1 U2 U3 U4]) + *P*([D1 U2 D3 D4]) + *P*([D1 U2 D3 N4]).

By using a first order Markov Chain, *P*([D1 U2 D3 U4]), for example, is computed as follows:



where , ,  and . The values |*D*1|, |*D*1*U*2|, |*U*2|, |*U*2*D*3|, |*D*3|, |*D*3*U*4| correspond, respectively, to the number of occurrences of symbol D1, the number of transitions from D1 to U2, the number of occurrences of symbol U2, the number of transitions from D1 to U2, the number of occurrences of symbol D3 and the number of transitions from D3 to U4. The remainder conditional probabilities needed to compute *P*(*N*(1, *p*_*B*_)) are computed in a similar way.

When *missing values are considered as valid errors*, *N*(*e*, *p*_*B*_) is computed using the alphabet Σ '∪ *mv'*, where *mv *is the symbol used for missing value and each element *mv' *is obtained by concatenating *m *and one number in the range {1, ..., |*C*|}, that is, *mv' *= {*mv*} × {1, ..., |*C*|}.

When only *restricted errors *are allowed, *N*(*e*, *p*_*B*_) is not computed using all the symbols in Σ*'*. The allowed substitutions for each symbol in *p*_*B *_are the *z *neighbors, both to the left and to the right of Σ*'*[*p*] that are considered as valid errors, where *p *is the position of the symbol *p*_*B*_[*k*] in Σ*'*.

In the case of *e-CCC-Biclusters with sign-changes *we compute the statistical significance of *B*, using the *p*-value(*B*), by obtaining the probability of a random occurrence under *H*_0 _of the expression patterns in the *e*-Neighborhoods of the patterns in the *p*_*B *_and , *K *= |*I*|-1 times in *n *= |*R*| -1 independent trials. We compute *P*(*N*(*e, p*_*B*_) ∪ *N*(*e*, )) as follows:



where  and |*N*(*e*, *p*_*B*_)|, |*N*(*e*, )|, *N*(*e*, *p*_*B*_)[*i*] and *N *(*e*, ) [*i*] are, respectively, the number of patterns and the *i*th pattern in the *e*-Neighborhood of the pattern *p*_*B *_and , respectively.

In the case of *e-CCC-Biclusters with scaled patterns *we compute the statistical significance of *B*, using the *p*-value(*B*), computed by obtaining the probability of a random occurrence under *H*_0 _of the patterns in the *e*-Neighborhood of the pattern *p*_*B*_, and its scaled patterns, (*p*_*B*_)^↑ ^and (*p*_*B*_)^↓^, *k *= |*I*| - 1 times in *n *= |*R*| - 1 independent trials, where *I *is the number of genes in *B *and |*R*| is the total number of genes in the matrix.

We compute *P*(*N*(*e*, *p*_*B*_) ∪ *N*(*e*, (*p*_*B*_)^↑^)) ∪ *N*(*e*, (*p*_*B*_)^↓^)) as follows:



where *shift *∈ {1, ..., *K*}, and *K *is the value used in *e*-CCC-Biclustering with scaled patterns to shift the expression patterns *K *symbols up and down.

#### Similarity measure

In order to compute the similarity measure between two *e*-CCC-Biclusters, *B*_1 _= (*I*_1_, *J*_1_) and *B*_2 _= (*I*_2_, *J*_2_), we use the Jaccard Index. In this work, this score is used to measure the overlap between two *e*-CCC-Biclusters both in terms of genes and conditions and is defined as follows:



where *B*_11 _= {(*i*, *j*): (*i*, *j*) ∈ *B*_1 _∧ (*i*, *j*) ∈ *B*_2_}, *B*_10 _= {(*i*, *j*): (*i*, *j*) ∈ *B*_1 _∧ (*i*, *j*) ∉ *B*_2_}, and *B*_01 _= {(*i*, *j*): (*i*, *j*) ∉ *B*_1 _∧ (*i*, *j*) ∈ *B*_2_}, for the genes *i *∈ *I*_1 _∪ *I*_2 _and the conditions *j *∈ *J*_1 _∪ *J*_2_.

Similarly, the gene similarity and condition similarity can be computed, respectively, as follows:



Note that, in practice, and since |*B*_1_| = |*I*_1_| × |*J*_1_| and |*B*_2_| = |*I*_2_| × |*J*_2_|, the similarity score as defined above can be computed easily using the fact that |*B*_1 _∩ *B*_2_| = |*I*_1 _∩ *I*_2_| × |*J*_1 _∩ *J*_2_| and |*B*_1 _∪ *B*_2_| = |*B*_1_| + |*B*_2_| - |*B*_1 _∩ *B*_2_|.

## Results and discussion

In this section we present and discuss the results obtained when applying the proposed *e*-CCC-Biclustering algorithm to real time series gene expression data. We also compare the performance of the proposed algorithm to that of CCC-Biclustering [9]. We first describe the dataset used to test the ability of the algorithm to find biologically relevant expression patterns in real data and to perform the comparison with CCC-Biclustering. This dataset describes the transcriptional responses of *Saccharomyces cerevisiae *to heat stress. We then show an application of *e*-CCC-Biclustering to the discovery of transcriptional regulatory modules. Finally, we present the comparison with CCC-Biclustering. All the results presented are based on the analysis of Gene Ontology annotations obtained using the GOToolbox database [28], together with information about transcriptional regulations available in the YEASTRACT database [29].

### Dataset

We used a dataset from Gasch et al. [30], concerning the *Saccharomyces cerevisiae *response to heat shock. This dataset comprises seven different time points along the first hour of exposure to 37°C (0, 0, 0, 5, 15, 30 and 60 minutes) and corresponds to the experiment identified as "*heat shock 2" *in the original group of datasets described by the authors. Since the first three time points are replicates of the steady state, we computed an average of three replicates of time zero and used a dataset with five time points. From the original 6152 ORFS we removed those with missing values and the ones that no longer existed in SGD (Saccharomyces Genome Database). For the remaining 6142 genes we obtained the correspondence between ORFS and gene names using the YEASTRACT database [29]. Since both *e*-CCC-Biclustering and CCC-Biclustering work with a discretized matrix we have then discretized this dataset using the discretization technique proposed by Ji and Tan [20,31]. The discretized matrix *A *is obtained in two steps. In the first step, *A' *is transformed into an *A" *= |*R*| × (|*C*| - 1) matrix of variations (see Equation 1). Once matrix *A" *is generated, the final discretized matrix *A*, also with |*R*| rows and |*C*|- 1 columns, is obtained in a second step by binning the values of the transformed matrix considering a threshold *t *> 0.

(1)

The expression matrix *A' *was standardized to zero mean and unit standard deviation, gene by gene, before the discretization process, and the discretization threshold *t *was set to the value of the standard deviation (*t *= 1). We refer to this preprocessed and discretized dataset as **DiscretizedHeatShock**.

### Application of *e*-CCC-Biclustering to the identification of transcriptional regulatory modules

To assess the biological relevance of *e*-CCC-Biclusters in real data we applied *e*-CCC-Biclustering to the **DiscretizedHeatShock **dataset. We allowed only one error (*e *= 1) and considered only errors in the 1-neighborhood of the symbols in the alphabet Σ = {*D*, *N*, *U*}. Note that this corresponds to applying one of the *e*-CCC-Biclustering extensions we propose in this work (*e*-CCC-Biclustering with restricted errors). By restricting the errors to the 1-neighborhood of the symbols in the alphabet Σ = {*D*, *N*, *U*}, our goal is to avoid the impact of a poor choice of the discretization thresholds in the ability of the algorithm to find all genes with coherent expression patterns. As such, the errors *D *< - > *N *and *N *< - > *U *are allowed but the error *D *< - > *U *is not permitted.

With this settings, 1-CCC-Biclustering found 170 maximal non-trivial 1-CCC-Biclusters. For these 170 1-CCC-Biclusters we computed the *p*-value using the method described in the previous section. Only 47 1-CCC-Biclusters were considered as statistically significant, at the 1% level, after applying the Bonferroni correction for multiple testing. All the 1-CCC-Biclusters not passing this statistical test were discarded. The remainder 47 were then sorted in ascending order of the statistical *p*-value previously computed. See additional file 3: **1_ccc_biclusters **for a summary of these 47 *e*-CCC-Biclusters.

**Figure 13:**
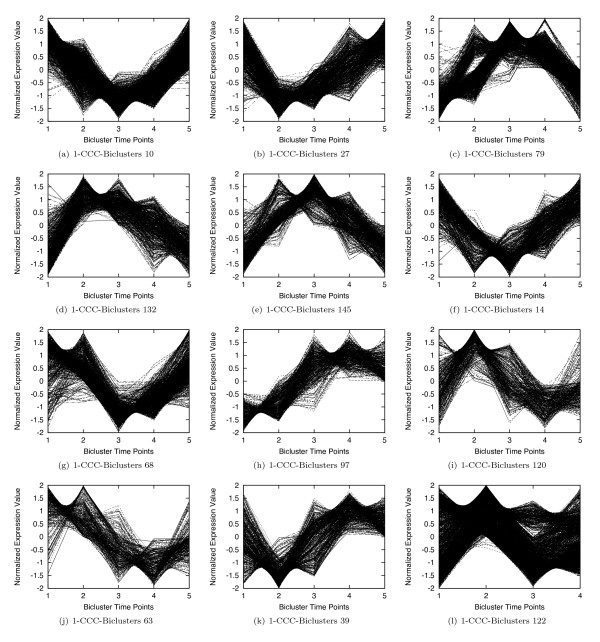
**Expression patterns of the 1-CCC-Biclusters surviving the overlapping filter**. This figure shows two types of expression patterns: transcriptional up-regulation (1-CCC-Biclusters 79, 132, 145, 97, 120 and 39) and transcriptional down-regulation patterns (1-CCC-Biclusters 10, 27, 14, 68, 63 and 122).

In order to avoid the analysis of highly overlapping 1-CCC-Biclusters, we computed the similarities between the sorted 1-CCC-Biclusters using the Jaccard similarity score and filtered the 1-CCC-Biclusters with similarity above 25%. The filtering process removed 35 of the 47 1-CCC-Biclusters originally selected. Figure 13 shows the expression patterns of the 12 1-CCC-Biclusters that remain.

These 12 highly significant and non-redundant 1-CCC-Biclusters were then analyzed using the Gene Ontology annotations using the GOToolbox database [28], together with information about transcriptional regulations available in the YEASTRACT database [29].

**Figure 14:**
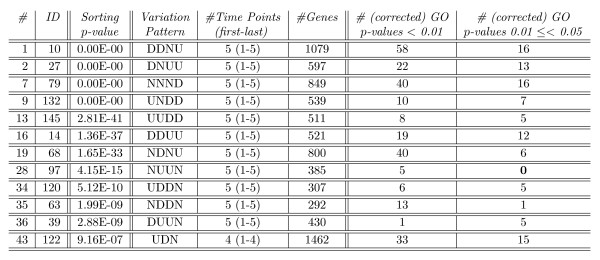
**Summary of the 1-CCC-Biclusters surviving the overlapping filter**. This table shows summary information about the 12 1-CCC-Biclusters surviving the overlapping filter. We show the statistical *p*-value of their expression patterns, the patterns themselves, the number of contiguous time points and the number of genes in each 1-CCC-Bicluster. Together with this information we present a summary of the results obtained when analyzing these 12 1-CCC-Biclusters using the Gene Ontology annotations restricted to those in the "Biological Process" ontology and above Level 2. We show the number of GO terms with highly significant and significant *p*-values, respectively, after Bonferroni correction for multiple testing. All 1-CCC-Biclusters are functionally enriched, having at least one term (several in general) whose *p*-value is highly statistical significant, after Bonferroni correction.

Figure 14 shows a summary of these top 12 1-CCC-Biclusters (expression patterns, number of genes and contiguous time points) together with information about functional enrichment relatively to terms in the Gene Ontology. To perform the analysis for functional enrichment, we considered only the "*Biological Process" ontology *and *terms above level 2*. We used the *p*-values obtained using the hypergeometric distribution to assess the over-representation of a specific GO term. In order to consider an *e*-CCC-Bicluster to be *highly significant*, we require its genes to show highly significant enrichment in one or more of the "Biological Process" ontology terms by having a Bonferroni corrected *p*-value below 0.01. An *e*-CCC-Bicluster is considered as *significant *if at least one of the GO terms analyzed is significantly enriched by having a (Bonferroni corrected) *p*-value in the interval [0.01, 0.05[. Note that, although we only consider as functionally enriched the terms with Bonferroni corrected *p*-values below 0.01 (for high statistical significance), or below 0.05 (for statistical significance), the *p*-values presented in the text are without correction, as it is common practice in the literature.

It is worth noting that all the 1-CCC-Biclusters analyzed have in general a large number of GO terms enriched (after Bonferroni correction), and all of them have at least one term whose *p*-value is highly significant (see Figure 14, for details). This means *all *the 1-CCC-Biclusters identified are biologically relevant as reported by functional enrichment analysis performed using the Gene Ontology.

**Figure 15:**
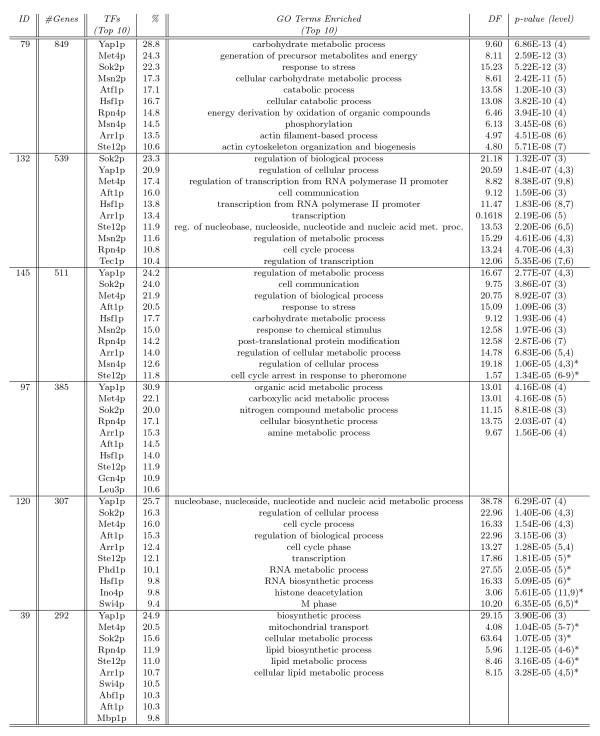
**GO terms and transcriptional regulations of the 1-CCC-Biclusters describing transcriptional up-regulation patterns**. This table shows a detailed analysis of the GO terms and transcriptional regulations of the 1-CCC-Biclusters describing transcriptional up-regulation patterns discovered by 1-CCC-Biclustering. When the set of genes in the 1-CCC-Bicluster has more than 10 transcription factors or more than 10 GO terms enriched, only the top 10 of each are shown. We only show the GO terms passing the Bonferroni correction for multiple testing at either the 1% level (highly significant) or the 5% level (significant). The *p*-values marked with * only passed the test at the 5% level. The *p*-values presented in the table are without correction as it is common practice in the literature.

**Figure 16:**
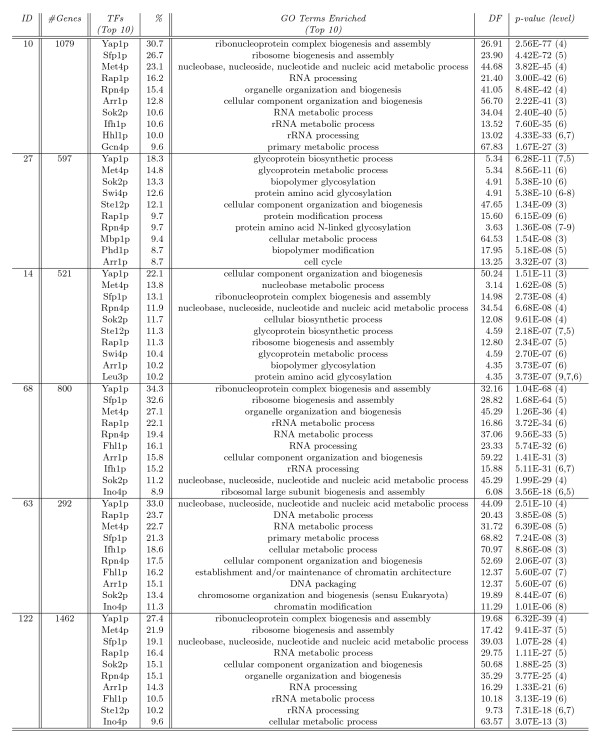
**GO terms and transcriptional regulations of the 1-CCC-Biclusters describing transcriptional down-regulation patterns**. This table shows a detailed analysis of the GO terms and transcriptional regulations of the 1-CCC-Biclusters describing transcriptional down-regulation patterns discovered by 1-CCC-Biclustering. When the set of genes in the 1-CCC-Bicluster has more than 10 transcription factors or more than 10 GO terms enriched, only the top 10 of each are shown. We only show the GO terms passing the Bonferroni correction for multiple testing at either the 1% level (highly significant) or the 5% level (significant). The *p*-values marked with * only passed the test at the 5% level. The *p*-values presented in the table are without correction as it is common practice in the literature.

Figure 15 and Figure 16 show a detailed analysis of the Gene Ontology annotations together with information about transcriptional regulations available in the YEASTRACT database, for the 1-CCC-Biclusters with transcriptional up-regulation patterns and 1-CCC-Biclusters with transcriptional down-regulation patterns, respectively. When the 1-CCC-Bicluster has more than 10 terms enriched or its genes are co-regulated by more than 10 transcription factors (TFs), only the 10 terms with lower *p*-value or the 10 transcription factors regulating the higher percentage of the genes in the 1-CCC-Bicluster are listed. The GO terms marked with * only passed the statistical test at the 5% level.

### Comparison with CCC-Biclustering: perfect versus approximate expression patterns

To assess the biological relevance of *e*-CCC-Biclusters in real data, and test our thesis regarding the potential superiority of this approach relatively to finding CCC-Biclusters with perfect expression patterns, we compared the results of *e*-CCC-Biclustering to those of CCC-Biclustering in the **DiscretizedHeatShock **dataset, as recently published by Madeira et al. [9].

In order to perform this comparison we reproduced the results in [9] using a prototype implementation of CCC-Biclustering coded in Java and made available by the authors in . We have also reproduced the biological analysis of CCC-Biclustering results since the data in the two databases (GoToolbox and YEASTRACT) used by the authors for this purpose was updated since the results in [9] were published.

Our intuition, when performing this comparison, is that allowing a small number of errors, per gene, in the perfect expression patterns identifying the CCC-Biclusters (*0*-CCC-Biclusters) discovered by CCC-Biclustering should improve the biological significance of the biclusters by considering genes with approximate expression patterns and thus minimizing the effect of possible discretization errors.

Note that, in the specific case of allowing 1 error in the pattern of a CCC-Bicluster one of the following three situations can happen: (1) the 1-CCC-Bicluster is equal to the CCC-Bicluster; (2) one or more genes, excluded from the CCC-Bicluster due to a single error are added to the 1-CCC-Bicluster; (3) the pattern of the *0*-CCC-Bicluster is extended (by adding one contiguous column at its beginning/end) leading to a 1-CCC-Bicluster with at least as many genes as the CCC-Bicluster and one additional contiguous column.

In this context, we believe the improvement in the biological significance of the results obtained by *e*-CCC-Biclustering should be two-fold:

1. The functional enrichment of the *e*-CCC-Biclusters should improve not only regarding the *p*-values of the GO terms enriched but also in terms of the number of GO terms enriched.

2. The number of genes regulated by relevant transcription factors in 1-CCC-Biclusters (TFs) should be higher than the number of genes regulated by the same TFs in the corresponding CCC-Biclusters.

The validation of the two points above will, in our opinion, demonstrate that *e*-CCC-Biclustering is not only able to recover genes with approximate expression patterns, that are potentially lost when only perfect expression patterns are considered, but also that the recovered genes are, in fact, biologically relevant to the problem under study.

**Figure 17:**
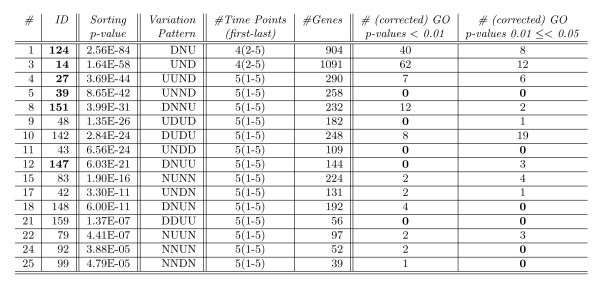
**CCC-Biclusters surviving the overlapping filter**. This table shows a summary of the CCC-Biclusters discovered by the CCC-Biclustering algorithm surviving the overlapping filter. It also shows the statistical *p*-value of their expression patterns, the patterns themselves, the number of contiguous time points and the number of genes in each CCC-Bicluster. Together with this information we present a summary of the results obtained when analyzing the 16 CCC-Biclusters using the Gene Ontology annotations restricted to those in the "Biological Process" ontology and terms above Level 2. We show the number of GO terms with highly significant and significant *p*-values, respectively, after Bonferroni correction for multiple testing. Several CCC-Biclusters are not functionally enriched after Bonferroni correction.

CCC-Biclustering discovered 167 maximal non-trivial CCC-Biclusters, which were then sorted in ascending order according to a statistical *p*-value similar to that we proposed here for *e*-CCC-Biclusters. From these only 25 CCC-Biclusters were considered as highly significant at the 1% level after applying the Bonferroni correction for multiple testing. In order to avoid the analysis of highly overlapping CCC-Biclusters, we have also computed the similarities between the sorted CCC-Biclusters using the Jaccard similarity score and filtered the CCC-Biclusters with similarity greater than 25%. The filtering process removed 9 of the 25 CCC-Biclusters originally selected. See additional file 4: **ccc_biclusters **for a summary of these 25 CCC-Biclusters. See also additional file 5: **1_ccc_biclusters_vs_ccc_biclusters **for a detailed comparison between the 47 highly significant 1-CCC-Biclusters discovered by 1-CCC-Biclustering restricted to errors in the 1-neighborhood of the symbols in the alphabet Σ = {*D*, *N*, *U*} and the 16 highly significant CCC-Biclusters found by CCC-Biclustering and analyzed by Madeira et al. [9]. It is clear from this table that most of the 47 1-CCC-Biclusters discovered by the 1-CCC-Biclustering algorithm are highly overlapping with one or more of the top 16 CCC-Biclusters identified by the CCC-Biclustering algorithm. Figure 17 shows a summary of the remaining 16 CCC-Biclusters analyzed according to the Gene Ontology (GO) annotations obtained using the GoToolBox [28], together with information about transcriptional regulations available in the YEASTRACT database [29], as performed above for 1-CCC-Biclustering results. See additional file 6: **ccc_biclusters_biological_validation **for a detailed analysis of the GO terms enriched and transcriptional regulations of these top 16 CCC-Biclusters.

Note that, unlike what happened with the top 12 1-CCC-Biclusters discovered by 1-CCC-Biclustering (Figure 14), the top 16 CCC-Biclusters discovered by CCC-Biclustering (Figure 17) have in general a small number of GO terms enriched (after Bonferroni correction), and several of them are not functionally enriched (after Bonferroni correction). This means some of the CCC-Biclusters identified by the CCC-Biclustering algorithm may not be biologically relevant according with the GO analysis.

**Figure 18:**
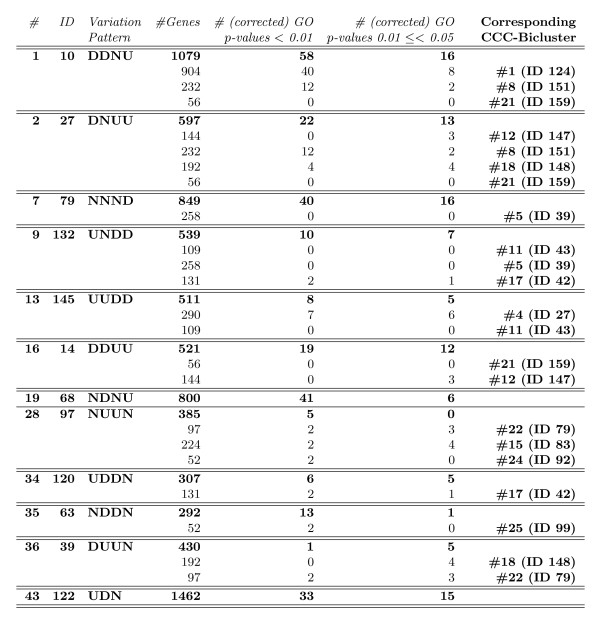
**Best CCC-Biclusters versus best 1-CCC-Biclusters**. This table shows the relationship between the top 12 1-CCC-Biclusters and the top 16 CCC-Biclusters. It is clear that, apart from two 1-CCC-Biclusters (IDs 68 and 122), all other 1-CCC-Biclusters correspond to the extension of one or several of the 16 CCC-Biclusters by adding genes with approximate expression patterns or extending the expression pattern of the CCC-Bicluster with a contiguous columns. All the resulting 1-CCC-Biclusters have a larger number of GO terms functionally enriched and are thus more relevant according to the functional enrichment analysis performed using the Gene Ontology.

Figure 18 shows the relationship between the top 12 1-CCC-Biclusters discovered by 1-CCC-Biclustering in Figure 14 (CCC-Biclusters with approximate patterns allowing one error per gene relatively to the expression pattern identifying the 1-CCC-Bicluster) and the top 16 CCC-Biclusters discovered by CCC-Biclustering in Figure 17 (CCC-Biclusters with perfect expression patterns). It is clear from this figure that, apart from two 1-CCC-Biclusters (IDs 68 and 122), all other 1-CCC-Biclusters correspond to the extension of one or several of the 16 CCC-Biclusters by adding genes with approximate expression patterns. The CCC-Bicluster with ID 124 was extended not only with genes with approximate patterns but also with a contiguous column at the left of its expression pattern.

It is worth noting that all the resulting 1-CCC-Biclusters have a larger number of GO terms functionally enriched. Moreover, even when the CCC-Biclusters are not functionally enriched, the 1-CCC-Biclusters obtained by considering approximate expression patterns instead of perfect patterns are *always *functionally enriched.

In order to show that the number of genes regulated by relevant TFs has increased in the 1-CCC-Biclusters when compared with the same number in the corresponding CCC-Biclusters, we used a set of relevant CCC-Biclusters chosen by Madeira et al. among the top 16 CCC-Biclusters in Figure 17. From these top 16 CCC-Biclusters the authors selected 6 CCC-Biclusters, which were then analyzed in more detail using the Gene Ontology annotations together with information about transcriptional regulation available in the YEASTRACT database. These *selected CCC-Biclusters *describe either transcriptional up-regulation (CCC-Biclusters with IDs 39, 27 and 14) or down-regulation patterns (CCC-Biclusters with IDs 147, 151 and 124). For these 6 CCC-Biclusters the authors identified relevant transcription factors (TFs) according to their expression pattern and relevant GO terms. For example, the heat-shock factor Hsf1p, together with the transcription factors Msn2p and Msn4p, known regulators of the general stress response in yeast, and the transcription factor Rpn4p, known stimulator of the proteosome genes, involved in the degradation of denatured or unnecessary proteins in stressed yeast cell [9], were identified by the authors as relevant TFs in CCC-Biclusters 39, 27 and 14. Note that apart from CCC-Bicluster 14, whose corresponding 1-CCC-Biclusters were removed during the application of the overlapping filter (see additional file 5: **1_ccc_biclusters_vs_ccc biclusters**), all these selected CCC-Biclusters have at least one corresponding 1-CCC-Bicluster in the top 12 (as it is also shown in Figure 18).

In this context, we decided to compare the number of genes regulated by each relevant TF identified in each of the selected CCC-Biclusters and the number of genes regulated by the same TFs in the corresponding 1-CCC-Biclusters. Remember that, if relevant genes (not included in these CCC-Biclusters due to a single error) were recovered and included in the corresponding 1-CCC-Biclusters, the number of genes regulated by relevant TFs should increase in the 1-CCC-Bicluster.

**Figure 19:**
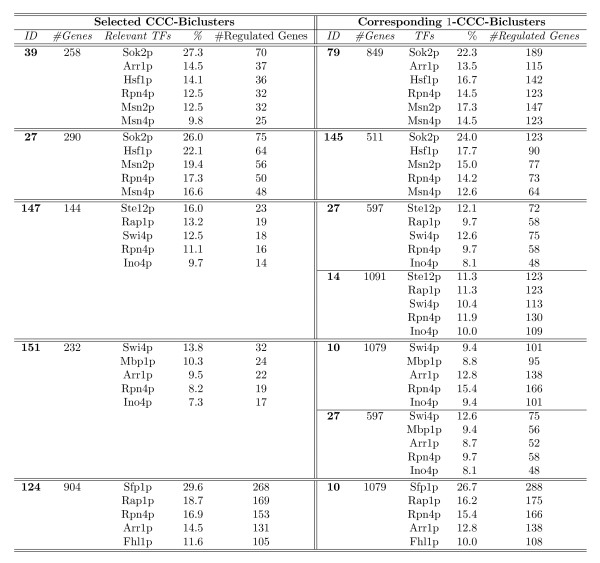
**Number of genes regulated by relevant TFs in selected CCC-Biclusters versus corresponding 1-CCC-Biclusters**. This table compares the number of genes regulated by the relevant TFs of the 5 selected CCC-Biclusters (CCC-Biclusters with IDs 39, 27, 147, 151 and 124) with the number of genes regulated by the same TFs in the corresponding 1-CCC-Biclusters. Note that these TFs might not appear in the top 10 in Figure 15 and Figure 16. It is clear from this table that the number of regulated genes by relevant TFs always increases in the corresponding 1-CCC-Biclusters.

Figure 19 shows, for each of the 5 selected CCC-Biclusters considered (CCC-Biclusters with IDs 39, 27, 147, 151 and 124), the set of relevant transcription factors together with the number of regulated genes and compares these numbers with those obtained for the same TFs in the corresponding 1-CCC-Bicluster(s). It is clear from this figure that the number of genes regulated by relevant TFs always increases in the corresponding 1-CCC-Biclusters. These results support our idea that *e*-CCC-Biclustering is able to recover genes with relevant expression patterns, that were missed due to small errors, and are in fact, biologically relevant to the problem under study. For example, in CCC-Bicluster 39, the relevant transcription factors Sok2p, Arr1p, Hsf1p, Rpn4p and Msn2p, regulated, respectively, 70, 37, 36, 32 and 32 of the 258 genes in the CCC-Bicluster. In the corresponding 1-CCC-Bicluster (ID 79) with 849 genes, these key transcription factors regulate, respectively, 189, 115, 142, 123 and 147 genes.

These results demonstrate that allowing the discovery of CCC-Biclusters with approximate patterns (*e*-CCC-Biclusters), rather than restricting the analysis to CCC-Biclusters with perfect expression patterns, can in fact improve the biological significance of the obtained results. These results also show, the superiority of the proposed *e*-CCC-Biclustering, algorithm, when compared with the CCC-Biclustering approach, in the identification of biologically relevant temporal patterns of expression.

## Conclusion and future work

In this work we proposed *e*-CCC-Biclustering, a new biclustering algorithm specifically developed for time series gene expression data analysis, that finds and reports all maximal contiguous column coherent biclusters with approximate expression patterns in time polynomial in the size of the expression matrix. These approximate patterns allow a given number of errors, per gene, relatively to an expression profile representing the expression pattern in the *e*-CCC-Bicluster. We described the algorithmic details of *e*-CCC-Biclustering, analyzed its computational complexity, and proposed extensions to improve the ability of the algorithm to discover other relevant expression patterns by being able to deal with missing values and allowing anticorrelated and scaled expression patterns. We also discussed different ways to compute the errors allowed in the approximate expression patterns. Finally, we described a scoring criterion based on a statistical test, used to sort *e*-CCC-Biclusters by increasing value of the probability that they have appeared by a random coincidence of events. Coupled with a similarity measure, used to filter highly overlapping *e*-CCC-Biclusters, this scoring criterion effectively identifies not only statistically but also biologically relevant *e*-CCC-Biclusters, which can then be useful to identify regulatory modules.

The results show the effectiveness of the approach and its relevance in the discovery of regulatory modules describing the transcriptomic expression patterns occurring in *Saccharomyces cerevisiae *in response to heat stress. Moreover, the comparison performed with a state of the art biclustering algorithm specifically developed for time series gene expression data analysis demonstrated the superiority of *e*-CCC-Biclustering in discovering statistically and biologically relevant temporal patterns of expression.

As short term future work, we plan to extend the algorithm to detected time-lagged regulations between genes and temporal patterns of expression in multiple time series gene expression matrices. The proposed algorithm can be easily extended to discover *e*-CCC-Biclusters with time-lags, enabling the discovery of important time-lagged regulations between genes, such as activation and inhibition, as well as temporal programs of expression, in which genes are activated one by one in a predefined order. Moreover, extending the algorithm to identify local temporal patterns of expression using multiple datasets should enable the discovery of conserved expression patterns and potentially help in the identification of common regulatory modules within and across-species. Our medium and long term research will be related with the use of the information about coherent expression patterns and co-regulation in the identification of regulatory modules, potentially helpful in the challenging area of inferring regulatory networks. This will require the development of efficient inference methods able to integrate heterogeneous data such as gene expression data, sequence data, and textual information scattered in scientific literature.

## Competing interests

The authors declare that they have no competing interests.

## Authors' contributions

SCM and ALO designed the *e*-CCC-Biclustering algorithm together with the proposed extensions and defined the scoring criterion for *e*-CCC-Biclusters based on the statistical significance of their expression patterns and similarity with other overlapping biclusters. SCM coded the prototype implementation of the algorithm in Java and wrote the first draft of the manuscript. SCM and ALO worked together towards the final version of the manuscript. All authors read and approved the final manuscript.

## Supplementary Material

Additional file 1 ***e*-CCC-Biclustering: Related work on biclustering algorithms for time series gene expression data**. Supplementary material describing related work on biclustering algorithms for time series gene expression data analysis. We describe in detail three state of the art biclustering approaches specifically designed to identify biclusters in gene expression time series and identify their strengths and weaknesses. We also explain and justify why we decided to compare the performance of *e*-CCC-Biclustering with that of CCC-Biclustering, but not with that of the *q*-clustering and CC-TSB algorithms. Click here for file

Additional file 2 ***e*-CCC-Biclustering: Algorithmic and complexity details**. Supplementary material describing algorithmic and complexity details of *e*-CCC-Biclustering. Click here for file

Additional file 3 **Highly significant 1-CCC-Biclusters**. Table showing a summary of the 47 1-CCC-Biclusters passing the Bonferroni correction for multiple testing at the 1% level when 1-CCC-Biclustering restricted to errors in the 1-neighborhood of the symbols in the alphabet Σ = {*D*, *N*, *U*} was applied to the **DiscretizedHeatShock **dataset. Click here for file

Additional file 4 **Highly significant CCC-Biclusters**. Table showing a summary of the 25 CCC-Biclusters passing the Bonferroni correction for multiple testing at the 1% level when CCC-Biclustering was applied to the **DiscretizedHeatShock **dataset. Click here for file

Additional file 5 **Highly significant 1-CCC-Biclusters *versus *highly significant CCC-Biclusters**. Table showing a comparison between the 47 highly significant 1-CCC-Biclusters discovered by 1-CCC-Biclustering restricted to errors in the 1-neighborhood of the symbols in the alphabet Σ = {*D*, *N*, *U*} and the 16 highly significant CCC-Biclusters found by CCC-Biclustering (after the applying the overlapping filter) and analyzed by Madeira et al. [9]. Both sets of biclusters were identified when the algorithm was applied to the **DiscretizedHeatShock **dataset. Click here for file

Additional file 6 **GO terms enriched and transcriptional regulations of the top 16 CCC-Biclusters**. Table showing a detailed analysis of the GO terms enriched and transcriptional regulations of the top 16 CCC-Biclusters discovered with CCC-Biclustering. When the set of genes in the CCC-Bicluster have more than 10 transcription factors or more than 10 GO terms enriched, only the top 10 of each are shown. We only show the GO terms passing the Bonferroni correction for multiple testing at either the 1% level (highly significant) or the 5% level (significant). The *p*-values marked with * only passed the test at the 5% level. The *p*-values presented in the table are without correction as it is common practice in the literature. Click here for file
