# Supplementary material for: A polynomial time biclustering algorithm for finding approximate expression patterns in gene expression time series
Source: Algorithms Mol Biol. 2009 Jun 4;4:8. doi: 10.1186/1748-7188-4-8 (PMC2709627; doi:10.1186/1748-7188-4-8)
Supplement: Additional file 1 — e-CCC-Biclustering: Related work on biclustering algorithms for time series gene expression data. Supplementary material describing related work on biclustering algorithms for time series gene expression data analysis. We describe in detail three state of the art biclustering approaches specifically designed to identify biclusters in gene expression time series and identify their strengths and weaknesses. We also explain and justify why we decided to compare the performance of e-CCC-Biclustering with that of CCC-Biclustering, but not with that of the q-clustering and CC-TSB algorithms. [file 1748-7188-4-8-S1.pdf]

e-CCC-Biclustering: Related work on biclustering algorithms for time series gene expression data

Sara C. Madeira*[1,2,3], Arlindo L. Oliveira[1,2]

[1] Knowledge Discovery and Bioinformatics (KDBIO) group, INESC-ID, Lisbon, Portugal
[2] Instituto Superior Técnico, Technical University of Lisbon, Lisbon, Portugal
[3] University of Beira Interior, Covilhã, Portugal

Email: Sara C. Madeira*- smadeira@kdbio.inesc-id.pt; Arlindo L. Oliveira - aml@inesc-id.pt;

*Corresponding author

Abstract

This document provides supplementary material describing related work on biclustering algorithms for time series gene expression data analysis. We describe in detail three state of the art biclustering approaches specifically design to discover biclusters in gene expression time series and identify their strengths and weaknesses.

Biclustering algorithms for time series gene expression data

Although many algorithms have been proposed to address the general problem of biclustering [1, 2], and despite the known importance of discovering local temporal patterns of expression, to our knowledge, only a few recent proposals have addressed this problem in the specific case of time series expression data [3–6]. These approaches fall into one of the following two classes of algorithms:

1. Exhaustive enumeration: CCC-Biclustering [5, 6] and *q*-clustering [4].

2. Greedy iterative search: CC-TSB algorithm [3].

These three approaches work with a single time series gene expression matrix and aim at finding biclusters defined as subsets of genes and subsets of **contiguous** conditions (time points) with coherent expression patterns. CCC-Biclustering and *q*-clustering work with a discretized version of the expression matrix while the CC-TSB-algorithm works with the original real-valued expression matrix.

In the next sections we describe in detail these three biclustering approaches and identify their strengths and weaknesses. We also justify why we decided to compare the performance of *e*-CCC-Biclustering with that of CCC-Biclustering, but not with that of the *q*-clustering and CC-TSB algorithms. The decision to exclude the last two algorithms from the comparisons is mainly based on existing analysis of these algorithms [6], and is basically related with complexity issues, in the case of *q*-clustering, and on poor results on real data obtained by the heuristic approach used by the CC-TSB algorithm.

CCC-Biclustering: Extracting all maximal CCC-Biclusters in linear time using a suffix tree

The *Contiguous Column Coherent Biclustering* (CCC-Biclustering) algorithm [5, 6], finds and reports all maximal CCC-Biclusters in time linear in the size of the expression matrix by manipulating a discretized version of the original expression matrix and using efficient string processing techniques based on suffix trees. The central idea of this approach is based on the relation between contiguous column coherent biclusters (CCC-Biclusters) and nodes in a generalized suffix tree constructed using Ukkonen's algorithm [7]. The authors demonstrate that, after performing a simple alphabet transformation, which appends the column number to each symbol in the matrix (as a preprocessing step in the algorithm), all nodes in the generalized suffix tree constructed with the set of strings corresponding to each row in the transformed matrix correspond to CCC-Biclusters. Since some nodes identify non-maximal CCC-Bicluster Madeira et al. introduce the concept of *maxNode* and use it to identify the maximal CCC-Biclusters with at least two rows (maximal CCC-Biclusters with only one one row are trivial and uninteresting from a biological point of view, and

are thus overlooked). A *maxNode* is defined as an internal node that, either does not have incoming suffix links, or in case it has incoming suffix links, these are only from nodes having a number of leaves in their subtree which is inferior to the number of leaves in the subtree of the *maxNode*. Using this definition the authors present and prove a theorem stating that, every maximal CCC-Bicluster with at least two rows corresponds to a maxNode in the generalized suffix tree, and each of these internal nodes defines a maximal CCC-Bicluster with at least two rows. This theorem is the base of CCC-Biclustering and the key to its linear time complexity.

Madeira et al. [6] also propose a statistical test to score the identified CCC-Biclusters, and to sort them by increasing value of the probability that they have appeared by a random coincidence of events, and a method to remove highly overlapping, and, therefore, redundant CCC-Biclusters. This scoring and filtering schema proved to be very effective in discovering relevant regulatory modules [6].

In this context, CCC-Biclustering has several strengths and is certainly a good choice when the goal is to discover statistical and biological relevant expression patterns in time series expression data: (1) efficiency in terms of computational complexity; (2) completeness (discovers all maximal CCC-Biclusters with perfect expression patterns); (3) possibility to use any discretization technique.

However, CCC-Biclustering does not consider approximate expression patterns, in the sense of allowing a given number of errors per gene in the expression pattern identifying the CCC-Bicluster. We believe this fact may limit its ability to discover other statistical and biologically relevant patterns. Moreover, and in our opinion, the significance of the CCC-Biclusters discovered can potentially be improved by considering genes with similar expression patterns, or just by extending the expression pattern representing the CCC-Biclusters by adding columns at left/right. Behind this reasoning are two facts: noise and measurement errors are inherent to most microarray experiments and small errors can potentially be introduced by the discretization process (due to poor choice of discretization thresholds or number of symbols). In the main manuscript we compare the performance of *e*-CCC-Biclustering with that of CCC-Biclustering and test our thesis that considering approximate expression patterns instead of perfect expression patterns can, in fact, improve the biological significance of the results.

The current version of CCC-Biclustering has also other small weaknesses, which are, however, easily overcomed as described in a recent technical report from the authors [8]. The algorithm does not deal directly with missing values (relying on a preprocessing step that either removes all genes with missing values or fills the missing values using one of the several input missing values techniques described in the literature); does not allow sign-changes in the expression patterns of the genes in CCC-Biclusters, thus

ignoring potential anticorrelation between genes; and also does not allow time-lag expression patterns thus ignoring potential delays in the activation of genes.

In the main manuscript we present extensions to e-CCC-biclustering to deal directly with missing values and identify scaled and anticorrelated expression patterns. We postpone the identification of e-CCC-Biclusters with time-lagged expression patterns to future work.

q-Clustering: Identifying time-lagged gene clusters in time series expression data

The q-Clustering algorithm [4] works with a discretized version of a time series gene expression matrix. As in the biclustering algorithms proposed in this thesis, Ji and Tan are interested in finding biclusters with consecutive columns identified by an expression pattern formed by a set of contiguous symbols in a given alphabet. The algorithm has three phases, which are described next.

In *Phase 1 (matrix transformation)*, the original expression matrix is transformed into a "slope" matrix to reflect the changing tendency of the genes over time using a three symbol alphabet $\Sigma = \{-1, 0, 1\}$.

After the discretization performed in *Phase 1*, *Phase 2 (generation of q-clusters)* generates a set of q-clusters using the rows in the discretized matrix, which are now sequences of values -1, 0 and 1. Each q-cluster contains a set of genes sharing the same expression pattern over some q consecutive time points. As such, the authors aim at finding genes that share similar subsequences of length $(q-1)$, where q is a user-defined parameter. Each q-cluster has a unique identifier, called its *q-clusterID*. The q-clusters are generated as follows: for each row (gene) in the "slope" matrix, the authors apply a sliding window of length $(q-1)$. As each $(q-1)$ substring is examined, its *q-clusterID* is determined and the $(geneID, st)$ pairs are inserted into the corresponding q-cluster, where $geneID$ is the identifier of the gene and st is the position of the starting point of the sliding window that identifies the $(q-1)$ character substring.

Similarly to Cheng and Church [10], the authors point-out that a mean-squared residue (MSR) metric can be introduced in this phase to determine the quality of a bicluster, so that those with mean-squared residue smaller than a user-specified value are retained as a high-quality bicluster, while the rest can be discarded.

Phase 3 (generation of time-lagged co-regulated relationships between the genes/genes clusters) has four tasks. *Task 1* extracts *biclusters* from the q-clusters. The $(geneID, st)$ pairs in each q-cluster are first sorted by starting position, so that all $(GeneID, st)$ pairs with the same starting position st are grouped together. This step directly identifies the biclusters contained in each q-cluster, since in a q-cluster all genes with the same starting position share the same pattern under the same q conditions. In the remaining steps of *Phase*

3, some additional computations are performed in order to extract potential regulations from the identified biclusters and allow approximate expression patterns. As such, and in order to draw additional relationships among biclusters, the authors propose *Task 2* and *Task 3*, which can be carried out to identify the activation and inhibition regulations, respectively. In this context, *Task 2* deals with gene relationships within a q-cluster by comparing the starting positions of the biclusters obtained from the q-clusters and aims at finding *activation regulations*. Since the biclusters (within a q-cluster) with different starting positions share the same pattern, and according to Ji and Tan, there is a promising time-lagged activation co-regulation relationship between these biclusters. In particular, Ji and Tan state that, given two biclusters, the one with the smaller starting position is a potential activator of the bicluster with the larger starting position. The time-lag between the two activations is given by the difference in the starting positions. Following the same idea, *Task 3* attempts to find *inhibition regulations*. To do so they start by finding a pair of q-clusters with opposite patterns. Such a pair of q-clusters is, according to the authors, a promising inhibition pair (the authors consider that two patterns are opposite to one another if corresponding elements between the two patterns are either both '0' or else '1' and '-1', respectively). According to their reasoning, genes/biclusters of one q-cluster with a smaller start position may inhibit genes/biclusters of the other q-cluster with a larger start position [4].

Finally, in *Task 4*, Ji and Tan handle *approximate matching*. They consider that similar or opposite patterns with only one or two exceptional elements may still be regarded as interesting by some researchers and deal with this problem as follows: for each q-cluster, they allow changes to be made to certain positions of the pattern. The corresponding q-cluster with the changed pattern is a potential candidate for co-regulation. For inhibition regulation, they find the q-cluster that has an opposite pattern from the changed pattern.

The authors consider that, since in their approach "0" indicates no obvious increasing or decreasing changing tendency, the patterns with too many "0"s are not interesting enough to be investigated. As such, they use another user-specified parameter, *Maximum Zero*, to control the maximum number of "0"s allowed in interesting patterns.

If appropriately implemented, q-Clustering would generate exactly the same biclusters as the ones generated by CCC-Biclustering (together with a set of non-maximal biclusters), when the same discretization technique is used and q-Clustering is executed until *Step 1* of *Phase 3* with q in the range of 2 to $|C|$. An implementation that uses the ideas of the Rabin-Karp string matching algorithm [?] would be able to perform this step in $O(|R||C|^2)$, although the implementation made available by the authors in [9] has complexity that is exponential on $|C|$. For these reasons we do not provide any experimental comparison between

e-CCC-Biclustering and q-Clustering.

Besides its high computational complexity, q-Clustering has a number of other weaknesses. The algorithm relies on a particular three symbol discretization matrix making impossible to use other discretization techniques more suitable to the dataset under study, or even discretization techniques using more symbols. Since the algorithm generates a large number of patterns (not necessarily maximal), the authors propose two filtering steps, which can be used along the algorithm to filter low quality biclusters: (1) the mean squared residue score (MSR) proposed by Cheng and Church [10] and the Maximum Zero parameter. This parameter controls the amount of symbols "0" allowed in the expression pattern of each q-cluster and is used in an attempt to filter patterns with poor changing tendency. However, having more than a predefined number of "0"s does not necessarily mean that the pattern is uninteresting. Moreover, since the expression patterns in q-clusters are found using a discretized matrix, we believe a statistical score could be used directly to evaluate the significance of these patterns and used to discard irrelevant patterns. This is proven to work well with CCC-Biclustering [6].

We highlight that approximate matching is not performed directly and appears here as a post-processing step that can be used to add more genes to previously discovered biclusters, which are also mined indirectly by analyzing the set of q-clusters. This means the authors are extending biclusters with perfect patterns by adding genes with similar patterns in order to obtain biclusters with approximate patterns. This procedure will certainly not return all possible biclusters with approximate patterns thus meaning q-clustering is not complete (even when the length of the expression patterns discovered is not limited by the value of the parameter q, as proposed by the authors, and ranges from 2 to $|C|$). In the main manuscript we show that e-CCC-Biclustering is complete and discovers all maximal contiguous columns coherent biclusters in polynomial time.

CC-TSB algorithm: Revealing co-regulated genes using a time-series biclustering algorithm

Zhang et al. [3] proposed the *Time-Series Biclustering algorithm* (CC-TSB algorithm), which modifies the heuristic algorithm of Cheng and Church [10], by restricting it to add and/or remove only columns that are contiguous to the partially constructed bicluster, thus forcing the resulting bicluster to have only contiguous columns. A predefined number of biclusters with contiguous columns is identified.

The CC-TSB algorithm contains two major steps: an iterative deletion procedure and an iterative insertion procedure. The output of the algorithm is a submatrix, which represents a bicluster. The first time the algorithm is executed, the submatrix under study is the entire gene expression matrix. The algorithm then

removes rows (genes) and columns (time points) from the submatrix, with the objective of minimizing the mean squared residue (MSR) [10] of the resulting submatrix. A row (gene) is removed from the submatrix if its expression profile is significantly different from others in the submatrix, measured by the ratio of the MSR computed for the elements in the row to that of the whole submatrix. If the ratio is larger than α, an empirically-chosen threshold, the row (gene) is removed. Columns (time points) are removed from the submatrix in a similar manner. To ensure that the time points in a bicluster are always consecutive, only the first and the last columns in the submatrix can be deleted. The deletion process terminates when the MSR of the resulting bicluster is below the upper limit δ.

Since some previously-deleted genes may be partially co-expressed with genes contained in the resulting submatrix, in the time interval of the submatrix, the algorithm then tries to recover these genes and insert them back into the submatrix in order to maximize the size of the bicluster discovered. The insertion operation is also performed for time points. The criterion for insertion is similar to the reverse of that for deletion: if the MSR of a row to that of the submatrix is less than α, the gene corresponding to the row is inserted into the bicluster. Due to the requirement for contiguity in the columns, only those next to the border of the submatrix are considered for column insertion.

Multiple biclusters are identified (as in the original Cheng and Church proposal) by masking the biclusters found so far with random values and using the new matrix in the next execution of the algorithm.

Due to its heuristic nature, this approach is not guaranteed to find the optimal set of biclusters. Moreover, its complexity is at least $\Omega(n|R|^2|C|)$, where n is the predefined number of biclusters to be discovered, and thus CCC-Biclustering is at least a factor of $\Theta(|R|)$ times faster. However, since this approach does not use discretization as a preprocessing step, Madeira et al. [6] have recently compared CCC-Biclustering with CC-TSB algorithm [3] using the same dataset and parameters used by Zhang et al.. The results obtained show the weakness of CC-TSB algorithm when applied to real time series gene expression data. The heuristic method proposed by Zhang et al. is not effective in finding interesting biclusters with contiguous columns in gene expression time series since the restriction imposed on the columns that can be removed makes the algorithm converge rapidly to a local minimum, from which it does not escape [6]. This is in part due to the fact that once one large coherent bicluster is discovered, the masking process corrupts the data making it difficult to find other interesting biclusters. In this context, Madeira et al. show that this method converges to biclusters with a high number of columns, which are, in most cases, all the columns in the dataset (meaning this algorithm is in fact looking for gene clusters, and not biclusters, which makes it useless for the purposes of identifying local patterns) and whose MSR values are not optimal. Besides this fact, Madeira et al. show

that, the statistical significance test coupled with CCC-Biclustering to score the set of CCC-Biclusters is able to find highly significant expression patterns shared by a relatively large number of genes with a small MSR, thus proving the superiority of CCC-Biclustering when compared to CC-TSB algorithm in finding relevant biclusters with contiguous columns in time series expression data.

For these reasons, we will not compare *e*-CCC-Biclustering against CC-TSB algorithm in the main manuscript, although we will perform a comparison with CCC-Biclustering.

References

1. Mechelen IV, Bock HH, Boeck PD: **Two-mode clustering methods: a structured overview**. *Statistical Methods in Medical Research* 2004, **13**(5):979–981.
2. Madeira SC, Oliveira AL: **Biclustering algorithms for biological data analysis: a survey**. *IEEE/ACM Transactions on Computational Biology and Bioinformatics* 2004, **1**:24–45.
3. Zhang Y, Zha H, Chu CH: **A Time-Series Biclustering Algorithm for Revealing Co-Regulated Genes**. In *Proc. of the 5th IEEE International Conference on Information Technology: Coding and Computing* 2005:32–37.
4. Ji L, Tan K: **Identifying time-lagged gene clusters using gene expression data**. *Bioinformatics* 2005, **21**(4):509–516.
5. Madeira SC, Oliveira AL: **A Linear Time Biclustering Algorithm for Time Series Gene Expression Data**. In *Proc. of 5th Workshop on Algorithms in Bioinformatics*, Springer Verlag, LNCS/LNBI 3692 2005:39–52.
6. Madeira SC, Teixeira MC, Sá-Correia I, Oliveira AL: **Identification of Regulatory Modules in Time Series Gene Expression Data using a Linear Time Biclustering Algorithm**. *IEEE/ACM Transactions on Computational Biology and Bioinformatics, 21 Mar 2008. IEEE Computer Society Digital Library. IEEE Computer Society, 24 March 2008*[http://doi.ieeecomputersociety.org/10.1109/TCBB.2008.34].
7. Ukkonen E: **On-line construction of suffix trees**. *Algorithmica* 1995, **14**:249–260.
8. Madeira SC, Gonçalves JP, Oliveira AL: **Efficient Biclustering Algorithms for identifying transcriptional regulation relationships using time series gene expression data**. Tech. Rep. 22, INESC-ID 2007.
9. Ji L, Tan K: **Identifying time-lagged gene clusters using gene expression data - Supplementary information**. *http://www.comp.nus.edu.sg/~jiliping/p2.htm, [September 20, 2006].*
10. Cheng Y, Church GM: **Biclustering of Expression Data**. In *Proc. of the 8th International Conference on Intelligent Systems for Molecular Biology* 2000:93–103.
