# Supplementary material for: A polynomial time biclustering algorithm for finding approximate expression patterns in gene expression time series
Source: Algorithms Mol Biol. 2009 Jun 4;4:8. doi: 10.1186/1748-7188-4-8 (PMC2709627; doi:10.1186/1748-7188-4-8)
Supplement: Additional file 2 — e-CCC-Biclustering: Algorithmic and complexity details. Supplementary material describing algorithmic and complexity details of e-CCC-Biclustering. [file 1748-7188-4-8-S2.pdf]

e-CCC-Biclustering: Algorithmic and complexity details

Sara C. Madeira*[1,2,3], Arlindo L. Oliveira[1,2]

[1] Knowledge Discovery and Bioinformatics (KDBIO) group, INESC-ID, Lisbon, Portugal
[2] Instituto Superior Técnico, Technical University of Lisbon, Lisbon, Portugal
[3] University of Beira Interior, Covilhã, Portugal

Email: Sara C. Madeira*- smadeira@kdbio.inesc-id.pt; Arlindo L. Oliveira - aml@inesc-id.pt;

*Corresponding author

Abstract

This document provides supplementary material describing algorithmic and complexity details of e-CCC-Biclustering. For clarity we repeat here the details of the main steps of e-CCC-Biclustering already presented in the main manuscript. We believe that a complete description of the algorithmic details makes it easier to understand the detailed complexity analysis presented afterwards.

e-CCC-Biclustering: Algorithmic details

In this section we perform a detailed algorithmic analysis of each of the 4 steps of e-CCC-Biclustering presented in Algorithm 1:

1. Reporting all right maximal e-CCC-Biclusters: procedure `computeRightMaximalBiclusters`.
2. Deleting valid models not corresponding to left-maximal e-CCC-Biclusters: procedure `deleteNonLeftMaximalBiclusters`.
3. Deleting valid models representing the same e-CCC-Biclusters: procedure `deleteRepeatedBiclusters`.
4. Reporting all maximal e-CCC-Biclusters: procedure `reportMaximalBiclusters`.

Algorithm 1: e-CCC-Biclustering

Input : A, Σ, e, q_r, q_c
Output: Maximal e-CCC-Biclusters.

```
{S_1, ..., S_|R|} ⟵ alphabetTransformation(A, Σ)
modelsOcc ⟵ {}/* List of (m, genesOcc_m, numberOfGenesOcc_m)    */
computeRightMaximalBiclusters(Σ, e, q_r, q_c, {S_1, ..., S_|R|}, modelsOcc)
deleteNonLeftMaximalBiclusters(modelsOcc)
if e > 0 then
    deleteRepeatedBiclusters(modelsOcc)
reportMaximalBiclusters(modelsOcc)
```

1. Computing valid models corresponding to right-maximal e-CCC-Biclusters

Procedure `computeRightMaximalBiclusters`

Input: Σ, e, q_r, q_c, $\{S_1, \ldots, S_{|R|}\}$, $modelsOcc$
/* The value of $modelsOcc$ is updated. */
$T_{right} \longleftarrow$ constructGeneralizedSuffixTree($\{S_1, \ldots, S_{|R|}\}$)
addNumberOfLeaves(T_{right}) /* Adds $L(v)$ to each node v in T_{right}. */
if $e \neq 0$ **then**
  addColorArray(T_{right})
  /* Adds $colors_v$ to every node v in T_{right}: $colors_v[i] = 1$, if there is a leaf in the subtree rooted at v that is a suffix of S_i; $colors_v[i] = 0$, otherwise. */
$m \longleftarrow$ "" /* model m is a string $[m[1] \ldots m[length_m]]$ */
$length_m \longleftarrow 0$
$father_m \longleftarrow$ "" /* $father_m$ is a string $[m[1] \ldots m[length_m - 1]]$ */
$numberOfGenesOcc_{father_m} \longleftarrow 0$
$Occ_m \longleftarrow \{\}$ /* List of node-occurrences (v, v_{err}, p) */
addNodeOccurrence(Occ_m, (root(T_{right}), 0, 0))
$Ext_m \longleftarrow \{\}$ /* Ext_m is the set of possible symbols α to extend the model m. */
if $e = 0$ **then**
  forall *edges* $E(v_i)$ *leaving from node* root(T_{right}) *to a node* v_i **do**
    if $label(E(v_i))[1]$ *is not a string terminator* **then**
      addSymbol(Ext_m, $label(E(v_i))[1]$)
else
  forall *symbols in* Σ' **do**
    /* Σ' must be in lexicographic order. */
    addSymbol(Ext_m, $\Sigma'[i]$)
$length_m \longleftarrow 0$
spellModels(Σ,e,q_r,q_c,$modelsOcc$,T_{right},m,$length_m$,Occ_m,Ext_m,$father_m$,$numberOfGenesOcc_{father_m}$)

Below we present the algorithmic details of the recursive procedure `spellModels`, which is an adaption of SPELLER [1] able to identify all valid models corresponding to right-maximal e-CCC-Biclusters. These e-CCC-Biclusters are identified by right-maximal valid models (patterns that cannot be extended without losing occurrences) and their corresponding node-occurrences (patterns starting and finish at the same time points in a set of rows in the time series expression matrix).

Procedure `spellModels`

/* Called recursively. Stores right-maximal e-CCC-Biclusters in *modelsOcc*. */

Input : Σ, e, q_r, q_c, *modelsOcc*, T_{right}, m, $length_m$, Occ_m, Ext_m, $father_m$, $numberOfGenesOcc_{father_m}$

/* The value of *modelsOcc* is updated. */

keepModel(q_r, q_c, *modelsOcc*, T_{right}, m, $length_m$, Occ_m, $father_m$, $numberOfGenesOcc_{father_m}$)
if $length_m \leq |C|$ **then**
  /* $|C|$ is the length of the longest model */
  forall *symbols* α *in* Ext_m **do**
    if α *is not a string terminator* **then**
      maxGenes $\longleftarrow$ 0 /* Sum of $L(v)$ for all node-occurrences (v,v_{err},p) in $Occ_{m\alpha}$ */
      minGenes $\longleftarrow$ ∞ /* Minimum $L(v)$ in all node-occurrences (v,v_{err},p) in $Occ_{m\alpha}$ */
      $Colors_{m\alpha} \longleftarrow \{\}$
      if $e > 0$ **then**
        $Colors_{m\alpha}[i] \leftarrow 0, 1 \leq i \leq |R|$
        /* $colors_{m\alpha}[i] = 1$, if there is a node-occurrence of m in S_i; */
        /* $colors_{m\alpha}[i] = 0$, otherwise */
      $Ext_{m\alpha} \longleftarrow \{\}$
      $Occ_{m\alpha} \longleftarrow \{\}$
      forall *node-occurrences* (v, v_{err}, p) *in* Occ_m **do**
        /* If p = 0 we are at node v. Otherwise, we are at edge $E(v)$ between nodes $father(v)$ and v at point $p > 0$. */
        if $p = 0$ **then**
          extendFromNodeWithoutErrors(Σ, e, T_{right}, (v, v_{err}, p), m, α, $Occ_{m\alpha}$, $Colors_{m\alpha}$, $Ext_{m\alpha}$, *maxGenes*, *minGenes*)
          if ($v_{err} < e$) **then**
            extendFromNodeWithErrors(Σ,e, T_{right}, (v, v_{err}, p), m, α, $Occ_{m\alpha}$, $Colors_{m\alpha}$, $Ext_{m\alpha}$, *maxGenes*, *minGenes*)
        else
          extendFromEdgeWithoutErrors(T_{right}, Σ, e, (v, v_{err}, p), m, α, m, $Occ_{m\alpha}$, $Colors_{m\alpha}$, $Ext_{m\alpha}$, *maxGenes*, *minGenes*)
          if $x_{err} < e$ **then**
            extendFromEdgeWithErrors(Σ, e, T_{right}, (v, v_{err}, p), m, α, $Occ_{m\alpha}$, $Colors_{m\alpha}$, $Ext_{m\alpha}$, *maxGenes*, *minGenes*)
      if modelHasQuorum(*maxGenes*, *minGenes*, $Colors_{m\alpha}$, q_r) **then**
        spellModels(Σ, e, q_r, q_c, *modelsOcc*, T_{right}, $m\alpha$, $length_m + 1$, $Occ_{m\alpha}$, $Ext_{m\alpha}$, $father_{m\alpha}$, $numberOfGenesOcc_m$)

Next we present the algorithmic details of the procedures and functions used in the procedure `spellModels` described above. We use the following order:

1. Procedure `keepModel` [called in `spellModels`]
2. Procedure `checkRightMaximality` [called in `keepModel()`]
3. Procedure `extendFromNodeWithoutErrors` [called in `spellModels`]
4. Procedure `extendFromNodeWithErrors` [called in `spellModels`]
5. Procedure `extendFromEdgeWithoutErrors` [called in `spellModels`]

6. Procedure `extendFromEdgeWithoutErrors` [called in `spellModels`]

7. Function `modelHasQuorum` [called in `spellModels`]

8. Procedure `extendModel` [called in `extendFromNodeWithoutErrors()`, `extendFromNodeWithErrors()`, `extendFromEdgeWithoutErrors` and `extendFromEdgeWithErrors()`]

Procedure `keepModel`

Input: q_r, q_c, $modelsOcc$, T_{right}, $length_m$, Occ_m, $father_m$, $numberOfGenesOcc_{father_m}$
/* The value of $modelsOcc$ is updated. */
/* The initial model corresponding to the root should never be kept. */
if $length_m > 0$ **then**
    /* Check columns quorum. */
    if $length_m \geq q_c$ **then**
        $genesOcc_m \longleftarrow$ `computeGenesInNodeOccurrences`(Occ_m, T_{right}) /* bit vector */
        $numberOfGenesOcc_m \longleftarrow$ `computeNumberOfGenesInNodeOccurrences`($genesOcc_m$)
        /* Check rows quorum. */
        if $numberOfGenesOcc_m \geq q_c$ **then**
            `addModelAndOccurrences`($modelsOcc$, (m, $genesOcc_m$, $numberOfGenesOcc_m$))
            /* Check if $father_m$ satisfied the columns quorum. If so it was kept. */
            if $length_{father_m} \geq q_c$ **then**
                /* Throw away $father_m$ if it does not correspond to a right-maximal e-CCC-Bicluster. */
                `checkRightMaximality`($modelsOcc$, $numberOfGenesOcc_m$, $father_m$, $numberOfGenesOcc_{father_m}$)

Procedure `checkRightMaximality`

Input: $modelsOcc$, $numberOfGenesOcc_m$, $father_m$, $numberOfGenesOcc_{father_m}$
/* The value of $modelsOcc$ is updated. */
/* Check if $father_m$ was the root. If so it was not kept. */
if $length_{father_m} \neq 0$ **then**
    if $numberOfGenesOcc_m = numberOfGenesOcc_{father_m}$ **then**
        `deleteModelAndOccurrences`($modelsOcc$, $father_m$)

Procedure `extendFromNodeWithoutErrors`

Input: Σ, e, m, $(v, v_{err}, 0)$, α, $Occ_{m\alpha}$, $Colors_{m\alpha}$, $Ext_{m\alpha}$, $maxGenes$, $minGenes$
/* The values of $Occ_{m\alpha}$, $Colors_{m\alpha}$, $Ext_{m\alpha}$, $maxGenes$, $minGenes$ are updated. */
forall *edges* $E(v')$ *leaving node* v *to a node* v' **do**
    if $label(E(v'))[1] = \alpha$ **then**
        /* m can be extended with α without errors */
        `extendModel`(Σ, e, m, $(v, v_{err}, 0)$, α, $Occ_{m\alpha}$, $Colors_{m\alpha}$, $Ext_{m\alpha}$, $maxGenes$, $minGenes$, $E(v')$, 0)

Procedure `extendFromNodeWithErrors`

```
Input: Σ, e, T_right, (v,v_err,0), α, Occ_mα, Colors_mα, Ext_mα, maxGenes, minGenes
/* The values of Occ_mα, Colors_mα, Ext_mα, maxGenes, minGenes are updated.   */
forall edges E(v') leaving node v to node v' do
    if label(E(v'))[1] ≠ α then
        if label(E(v'))[1] is not a string terminator then
            if v = root(T_Right) then
                /* Test if first symbol in label(E(v')) has column number equal to that of α.
                   C(label(E(v'))[1]) is the column number of symbol label(E(v'))[1]   */
                if C(label(E(v'))[1]) = C(α) then
                    extendModel(Σ, e, T_right, (v,v_err,0), α, Occ_mα, Colors_mα, Ext_mα, maxGenes,
                    minGenes, E(v'),1)
            else
                extendModel(Σ, e, T_right, (v, v_err, 0), α, Occ_mα, Colors_mα, Ext_mα, maxGenes, minGenes,
                E(v'),1)
```

Procedure `extendFromEdgeWithoutErrors`

```
Input: Σ, e, T_right, (v, v_err, p), α, Occ_mα, Colors_mα, Ext_mα, maxGenes, minGenes
/* The values of Occ_mα, Colors_mα, Ext_mα, maxGenes, minGenes are updated.   */
if label(E(v))[p + 1] = α then
    extendModel(Σ,e,T_right,(v,v_err,p),α,Occ_mα, Colors_mα,Ext_mα,maxGenes, minGenes,E(v),0)
```

Procedure `extendFromEdgeWithErrors`

```
Input: Σ, e, T_right, (v, v_err, p), α, Occ_mα, Colors_mα, Ext_mα, maxGenes, minGenes
/* The values of Occ_mα, Colors_mα, Ext_mα, maxGenes, minGenes are updated.   */
if label(E(v))[p + 1] ≠ α then
    if label(E(v))[1] is not a string terminator then
        extendModel(Σ,e,T_right,(v,v_err,p),α,Occ_mα,Colors_mα,Ext_mα,maxGenes,minGenes,E(v),1)
```

Function `modelHasQuorum`

```
Input  : maxGenes, minGenes, Colors_m, q_r
Output: True or false
if maxGenes < q_r then
    return false
else
    if e = 0 then
        return true
    else
        if minGenes ≥ q_r then
            return true
        else
            if cardinality(Colors_mα) ≥ q_r then
                return true
            else
                return false
```

Procedure `extendModel`

Input: Σ, e, T_{right}, (v, v_{err}, p), α, $Occ_{m\alpha}$, $Colors_{m\alpha}$, $Ext_{m\alpha}$, $maxGenes$, $minGenes$, $E(x)$, $extension_{err}$

/* The values of $Occ_{m\alpha}$, $Colors_{m\alpha}$, $Ext_{m\alpha}$, $maxGenes$, $minGenes$ are updated. */

if $p > 0$ **then**
 /* If $p = 0$: we at node v descending edge $E(x)$, where x is the node reached by descending edge $E(x)$ and thus $father_x = v$. */
 /* If $p > 0$: we are at point p descending edge $E(x)$. In this case, $E(x)$ is the edge from $father_v$ to v and thus $x = v$. */
 x = v

if $|label(E(x))| = p + 1$ **then**
 /* Extension of m with α will reach node x */
 /* Update $Occ_{m\alpha}$ */
 `addNodeOccurrence`($Occ_{m\alpha}$, (x, $v_{err} + extension_{err}$, 0))
 /* Update $maxGenes$ and $minGenes$ */
 $maxGenes \leftarrow maxGenes + L(x)$
 /* When $e = 0$, maxGenes = minGenes (the number of node-occurrences of $m\alpha$ = 1) */
 if $L(x) < minGenes$ **then**
  $minGenes \leftarrow L(x)$
 /* Update $Colors_{m\alpha}$ */
 if $e > 0$ **then**
  $Colors_{m\alpha} \leftarrow Colors_{m\alpha} \vee Colors_x$
 /* Update $Ext_{m\alpha}$ */
 if $v_{err} = e$ **then**
  /* Error limit reached */
  forall *edges* $E(x')$ *leaving node* x *to a node* x' **do**
   if $label(E(x'))[1]$ *is not a string terminator* **then**
    `addSymbol`($Ext_{m\alpha}, label(E(x'))[1]$)
 else
  /* Errors are still allowed. */
  /* $C(\alpha)$ is the column number of symbol α. */
  forall *symbols* s *in* $\Sigma_{C(\alpha)+1} = \{f(a, C(\alpha) + 1), \forall a \in \Sigma\}$ **do**
   `addSymbol`($Ext_{m\alpha}$, s)

else
 /* If $p = 0$: we will stay at edge $E(x)$ between nodes v and x at point $p = 1$. */
 /* If $p > 0$: we will stay at edge $E(v)$ between $father(v)$ and v at point $p + 1$. */
 /* Update $Occ_{m\alpha}$ */
 `addNodeOccurrence`($Occ_{m\alpha}$, (x, $v_{err} + extension_{err}$, $p + 1$))
 /* Update $maxGenes$ and $minGenes$ */
 $maxGenes \leftarrow maxGenes + L(x)$
 if $L(x) < minGenes$ **then**
  $minGenes \leftarrow L(x)$
 /* Update $Colors_{m\alpha}$ */
 if $e > 0$ **then**
  $Colors_{m\alpha} \leftarrow Colors_{m\alpha} \vee Colors_x$
 if $x_{err} = e$ **then**
  /* Error limit reached. */
  if $label(E(x))[p + 2]$ *is not a string terminator* **then**
   `addSymbol`($Ext_{m\alpha}$, $label(E(x))[p + 2]$)
 else
  /* Errors are still allowed. */
  /* $C(\alpha)$ is the column number of symbol α. */
  if $C(\alpha) + 1 \leq |C|$ **then**
   /* Extension will not be a string terminator. */
   forall *symbols* s *in* $\Sigma'_{C(\alpha)+1} = \{f(a, C(\alpha) + 1), \forall a \in \Sigma\}$ **do**
    `addSymbol`($Ext_{m\alpha}$, s)

2. Deleting valid models not corresponding to left-maximal e-CCC-Biclusters

Procedure `deleteNonLeftMaximalBiclusters`

Input: *modelsOcc*

/* The value of *modelsOcc* is updated. */

$T_{left} \longleftarrow$ `createTrie` ()

/* Array which will store references to nodes in T_{left} */

$R_{nodes} \longleftarrow \{\}$

foreach *model and occurrences (m, genesOcc$_m$, numberOfGenesOcc$_m$) in modelsOcc* **do**

  $m_r \longleftarrow$ `ReverseModel(`m`)`

  $nodeRepresentingModel \longleftarrow$ `addReverseModelToTrie(`T_{left}, m_r`)`

  /* Each node in T_{left} stores two integers: 1) the number of genes in the model it represents, $genes_v$ (0 if it does not represent the end of a model); and 2) the maximum number of genes in the subtree rooted at v, $maxGenes_{subtree_v}$ (computed later). Both these values are initialized with 0. */

  `addNumberOfGenes(`$nodeRepresentingModel$,$numberOfGenesOcc_m$`)`

  `addReferenceToNode(`R_{nodes}, $nodeRepresentingModel$`)`

forall *nodes v in T_{left}* **do**

  /* Performed using a depth-first search (dfs) */

  if $genes_v > 0$ **then**

    /* Node v represents a model and is potentially left-maximal. */

    Mark v as "left-maximal"

  else

    Mark v as "non left-maximal"

  Compute the maximum number of genes in the subtree rooted at v

foreach *node v in T_{left}* **do**

  /* Performed using a depth-first search (dfs) */

  if $genes_v > 0$ *and* $genes_v = maxGenes_{subtree_v}$ **then**

    Mark v as "non left-maximal"

$p_{modelsOcc} \longleftarrow 0$

foreach *model and occurrences (m, genesOcc$_m$, numberOfGenesOcc$_m$) in modelsOcc* **do**

  if $R_{nodes}[p_{modelsOcc}]$ *is marked as "non-left maximal"* **then**

    `deleteModelAndOccurrences(`$modelsOcc$, m`)`

  $p_{modelsOcc} \longleftarrow p_{modelsOcc} + 1$

3. Deleting valid models representing the same e-CCC-Biclusters

Procedure `deleteRepeatedBiclusters`

```
Input: modelsOcc
/* The value of modelsOcc is updated.                                        */
H ⟵ createHashTable()
foreach model and occurrences (m, genesOcc_m, numberOfGenesOcc_m) in modelsOcc do
    firstColumn_m = C(m[1])
    lastColumn_m = C(m[length_m])
    key ⟵ createKey(firstColumn, lastColumn, genesOcc_m)
    value ⟵ (firstColumn, lastColumn, genesOcc_m)
    if containsKey(H, key) then
        value_key ⟵ getValue(H, key)
        if value = value_key then
            /* H already has a value representing the same CCC-Bicluster         */
            deleteModelAndOccurrences(modelsOcc, m)
        else
            insertKeyValue(key, value)
    else
        insertKeyValue(key, value)
```

4. Reporting all maximal e-CCC-Biclusters

Procedure `reportMaximalBiclusters`

```
Input: modelsOcc
foreach model and occurrences (m, genesOcc_m, numberOfGenesOcc_m) in modelsOcc do
    firstColumn_m = C(m[1])
    lastColumn_m = C(m[length_m])
    print(m, firstColumn_m, lastColumn_m, genesOcc_m)
```

e-CCC-Biclustering: Detailed complexity analysis

In this section we perform a detailed complexity analysis of each of the 4 steps of e-CCC-Biclustering presented in Algorithm 1:

1. Reporting all right maximal e-CCC-Biclusters: procedure `computeRightMaximalBiclusters`.
2. Deleting valid models not corresponding to left-maximal e-CCC-Biclusters: procedure `deleteNonLeftMaximalBiclusters`.
3. Deleting valid models representing the same e-CCC-Biclusters: procedure `deleteRepeatedBiclusters`.
4. Reporting all maximal e-CCC-Biclusters: procedure `reportMaximalBiclusters`.

Given an $|R|$ by $|C|$ discretized gene expression matrix, we show that, the asymptotic complexity of the algorithm is $O(\max(|R|^2|C|^{1+e}|\Sigma|^e, |R||C|^{2+e}|\Sigma|^e))$, where e is the maximum number of allowed errors allowed per gene in the expression pattern defining each e-CCC-Bicluster. However, in most cases of interest $|R| >> |C|$ and the complexity becomes $O(|R|^2|C|^{1+e}|\Sigma|^e)$. When $e = 0$, CCC-Biclustering [2, 3] can be used to obtain $O(|R||C|)$.

Procedure `computeRightMaximalBiclusters`

Given a discretized matrix A with $|R|$ rows and $|C|$ columns the alphabet transformation performed using the procedure `alphabetTransformation` returns the set of strings $S_i = \{S_1, \ldots, S_{|R|}\}$ (each with length $|C|$) needed to compute the generalized suffix tree T_{right} in $O(|R||C|)$ time.

The generalized suffix tree T_{right} (for the set of $|R|$ strings S_i with length $|C|$) can be constructed in $O(|R||C|)$ using Ukkonen's algorithm [4] and standard data structures used in suffix trees since the increase in the alphabet size from $|\Sigma|$ to $|C||\Sigma|$ due to the alphabet transformation does not affect the $O(|R||C|)$ construction and manipulation of T_{right} [2,3].

Adding the number of leaves, $L(v)$, to each node v in T_{right} is performed by a depth-first search *dfs*, which is linear on the number of nodes in T_{right} and therefore takes $O(|R||C|)$ time.

Adding the color array, a bitset of length $|R|$ needed only when $e > 0$, to each of the $O(|R||C|)$ nodes in T_{right} takes $O(|R|^2|C|)$ time.

All the remaining operations before the call to procedure `spellModels` take constant time, except the initialization of Ext_m, which is $O(|C||\Sigma|)$.

Consider now the complexity of the procedure `spellModels` when $e > 0$.

The procedure `keepModel` is $O(|R|^2||C|)$. The function `computeGenesInNodeOccurrences` takes $O(|R|^2||C|)$ since the number of node-occurrences in Occ_m is bounded by the maximum number of nodes in T_{right}, $O(|R||C|)$, and the manipulation of the color arrays takes $O(|R|)$. Given the bit vector with the genes, the function `computeNumberOfGenesInNodeOccurrences` is $O(|R|)$: in order to compute the genes in the node-occurrences of a model m (returned as a bit vector, $genesOcc_m$), the procedure `computeGenesInNodeOccurrences` performs a bitwise or between the bit vectors $colors_v$ of all node-occurrences (v, v_{err}, p) of m in case $e > 0$, and uses the value of $L(v)$ when $e = 0$ (in this case Occ_m has only one node-occurrence). The function `computeNumberOfGenesInNodeOccurrences` is then used to compute the number of genes. All the remaining operations, including the procedure `checkRightMaximality` takes constant time.

Since the number of valid models is $O(|R||C||N(e, S_i)|)$, where $|N(e, S_i)| \leq |C|^e|\Sigma|^e$, since $|S_i| = |C|$, the manipulation of the color array takes $O(|R|)$ time per model and all other operations take constant time, `spellModels` is $O(|R|^2|C|^{1+e}|\Sigma|^e)$, when $e > 0$.

Lets now look at the case where $e = 0$. In this case, `computeGenesInNodeOccurrences` also takes $O(|R|)$. Although, each model has now only one node-occurrence, the *dfs* needed to get the genes and the creation of the bit vector to be returned are both $O(|R|)$. The procedure `computeNumberOfGenesInNodeOccurrences` is also $O(|R|)$, `checkRightMaximality` takes also constant time, the color array does not exist and $|N(e, S_i)| = 1$. As such, when $e = 0$, `spellModels` is $O(|R|^2|C|)$.

In this context, the complexity of `computeRightMaximalBiclusters` is bounded by the complexity of `spellModels`, and is thus $O(|R|^2|C|^{1+e}|\Sigma|^e)$. Note that, when $e = 0$ the linear time complexity of $O(|R||C|)$ achieved by the CCC-Biclustering algorithm [2,3] is better than the $O(|R|^2|C|)$ bound obtained with e-CCC-Biclustering.

Procedure `deleteNonLeftMaximalBiclusters`

Since the number of models in $modelsOcc$ is $O(|R||C|^{1+e}|\Sigma|^e)$ and the size of the models is $O(|C|)$, the trie T_{left} can be constructed and manipulated in $O(|R||C|^{2+e}|\Sigma|^e)$.

Reversing the model m and inserting the reversed model m_r in the trie T_{left}, performed using the procedures `reverseModel` and `addReverseModelToTrie` take $O(|C|)$ time each. Storing the number of genes in the node v in T_{left} identifying the last symbol in m_r and storing the reference to this node in the array R_{nodes} take constant time. This enables the construction of T_{left} with the information about the number of genes where m occurs in $O(|R||C|^{2+e}|\Sigma|^e)$.

Since the number of nodes in T_{left} is $O(|R||C|^{2+e}|\Sigma|^e)$ each node v with be initially marked as either "left-maximal" or "non left-maximal" and the maximum number of genes stored it its subtree, $maxGenes_{subtree_v}$,

can be computed in $O(|R||C|^{2+e}|\Sigma|^e)$ using a *dfs*.

The remaining nodes v representing "non left-maximal" models can then be marked using another *dfs* performed again in $O(|R||C|^{2+e}|\Sigma|^e)$ where a simple constant time comparison os performed between the values of $genes_v$ and $maxGenes_{subtree_v}$ stored in each node identifying a model ($genes_v > 0$).

Finally, for each of the $O(|R||C|^{1+e}|\Sigma|^e)$ models stored in $modelsOcc$ we just have to verify if its corresponding node v in T_{left} is marked as "non left maximal". This verification, performed using the reference to node v the stored in R_{nodes} at position $p_{modelsOcc}$, and the potential removal of the model from $modelsOcc$ are both performed in constant time.

In this context, the procedure `deleteNonLeftMaximalBiclusters` has a worst case complexity of $O(|R||C|^{2+e}|\Sigma|^e)$.

Procedure `deleteRepeatedBiclusters`

The complexity of `deleteRepeatedBiclusters`, depends only on the number of valid models (assuming the hashtable accesses work in essentially constant time), $O(|R||C|^{1+e}|\Sigma|^e)$, and the time needed to compute the hash function, $O(|R|)$, leading to a worst case complexity of $O(|R|^2|C|^{1+e}|\Sigma|^e)$.

Procedure `reportMaximalBiclusters`

Since the number of genes in $genesOcc_m$ is $O(|R|)$ and computing the first and last column column of m takes constant time, the reporting procedure `reportMaximalBiclusters` is $O(|R|^2|C|^{1+e}|\Sigma|^e)$.

References

1. Sagot MF: **Spelling approximate repeated or common motifs using a suffix tree**. In *Proc. of Latin'98*, Springer Verlag, LNCS 1380 1998:111–127.
2. Madeira SC, Oliveira AL: **A Linear Time Biclustering Algorithm for Time Series Gene Expression Data**. In *Proc. of 5th Workshop on Algorithms in Bioinformatics*, Springer Verlag, LNCS/LNBI 3692 2005:39–52.
3. Madeira SC, Teixeira MC, Sá-Correia I, Oliveira AL: **Identification of Regulatory Modules in Time Series Gene Expression Data using a Linear Time Biclustering Algorithm**. *IEEE/ACM Transactions on Computational Biology and Bioinformatics, 21 Mar 2008. IEEE Computer Society Digital Library. IEEE Computer Society, 24 March 2008*[http://doi.ieeecomputersociety.org/10.1109/TCBB.2008.34].
4. Ukkonen E: **On-line construction of suffix trees**. *Algorithmica* 1995, **14**:249–260.
