# Supplementary material for: A polynomial time biclustering algorithm for finding approximate expression patterns in gene expression time series
Source: Algorithms Mol Biol. 2009 Jun 4;4:8. doi: 10.1186/1748-7188-4-8 (PMC2709627; doi:10.1186/1748-7188-4-8)
Supplement: Additional file 3 — Highly significant 1-CCC-Biclusters. Table showing a summary of the 47 1-CCC-Biclusters passing the Bonferroni correction for multiple testing at the 1% level when 1-CCC-Biclustering restricted to errors in the 1-neighborhood of the symbols in the alphabet Σ = {D, N, U} was applied to the DiscretizedHeatShock dataset. [file 1748-7188-4-8-S3.pdf]

Highly significant 1-CCC-Biclusters

#	*ID*	*Sorting p-value*	*Variation Pattern*	*#Time Points (first-last)*	*#Genes*
1	10	0.00E-00	DDNU	5 (1-5)	1079
2	27	0.00E-00	DNUU	5 (1-5)	597
3	45	0.00E-00	DDU	4 (2-5)	1264
4	49	0.00E-00	DNU	4 (2-5)	1467
5	53	0.00E-00	DUU	4 (2-5)	1356
6	64	0.00E-00	NDDU	5 (1-5)	670
7	79	0.00E-00	NNND	5 (1-5)	849
8	92	0.00E-00	NUND	5 (1-5)	1151
9	132	0.00E-00	UNDD	5 (1-5)	539
10	149	0.00E-00	UUND	5 (1-5)	1142
11	161	0.00E-00	UND	4 (2-5)	1803
12	136	8.31E-42	UNND	5 (1-5)	665
13	145	2.81E-41	UUDD	5 (1-5)	511
14	157	6.05E-41	UDD	4 (2-5)	1538
15	165	1.04E-38	UUD	4 (2-5)	1577
16	14	1.36E-37	DDUU	5 (1-5)	521
17	67	1.19E-35	NDNN	5 (1-5)	754
18	96	2.05E-35	NUUD	5 (1-5)	765
19	68	1.65E-33	NDNU	5 (1-5)	800
20	48	1.28E-29	DNN	4 (2-5)	1298
21	72	5.34E-29	NDUU	5 (1-5)	646
22	105	2.09E-24	NND	4 (2-5)	1579
23	26	6.43E-21	DNUN	5 (1-5)	452
24	125	9.88E-20	UDNU	5 (1-5)	750
25	19	1.45E-17	DNDU	5 (1-5)	614
26	153	8.31E-17	UUUD	5 (1-5)	475
27	22	2.30E-16	DNNN	5 (1-5)	515
28	97	4.15E-15	NUUN	5 (1-5)	385
29	88	1.07E-14	NUDD	5 (1-5)	702
30	6	1.20E-14	DDDU	5 (1-5)	427
31	9	1.10E-11	DDNN	5 (1-5)	454
32	61	4.72E-11	NDD	4 (1-4)	909
33	131	3.27E-10	UND	4 (1-4)	749
34	120	5.12E-10	UDDN	5 (1-5)	307
35	63	1.99E-09	NDDN	5 (1-5)	292
36	39	2.88E-09	DUUN	5 (1-5)	430
37	24	1.21E-08	DNU	4 (1-4)	866
38	148	2.27E-08	UUN	4 (1-4)	1434
39	34	1.13E-07	DUND	5 (1-5)	936
40	80	1.77E-07	NNNN	5 (1-5)	301
41	69	4.02E-07	NDU	4 (1-4)	1191
42	78	8.94E-07	NNN	4 (1-4)	1224
43	122	9.16E-07	UDN	4 (1-4)	1462
44	7	1.12E-06	DDN	4 (1-4)	1321
45	91	1.23E-06	NUN	4 (1-4)	1335
46	95	2.02E-06	NUU	4 (1-4)	1125
47	75	3.62E-05	NNDD	5 (1-5)	198
