# Supplementary material for: A polynomial time biclustering algorithm for finding approximate expression patterns in gene expression time series
Source: Algorithms Mol Biol. 2009 Jun 4;4:8. doi: 10.1186/1748-7188-4-8 (PMC2709627; doi:10.1186/1748-7188-4-8)
Supplement: Additional file 4 — Highly significant CCC-Biclusters. Table showing a summary of the 25 CCC-Biclusters passing the Bonferroni correction for multiple testing at the 1% level when CCC-Biclustering was applied to the DiscretizedHeatShock dataset. [file 1748-7188-4-8-S4.pdf]

Highly significant CCC-Biclusters

#	*ID*	*Sorting p-value*	*Variation Pattern*	*#Time Points (first-last)*	*#Genes*
1	124	2.56E-84	DNU	4 (2-5)	904
2	107	1.59E-75	NDNU	5 (1-5)	518
3	14	1.64E-58	UND	4 (2-5)	1091
4	27	3.69E-44	UUND	5 (1-5)	290
5	39	8.65E-42	UNND	5 (1-5)	258
6	163	1.07E-39	DDNU	5 (1-5)	260
7	84	2.91E-38	NUND	5 (1-5)	568
8	151	3.99E-31	DNNU	5 (1-5)	232
9	48	1.35E-26	UDUD	5 (1-5)	182
10	142	2.84E-24	DUDU	5 (1-5)	248
11	43	6.56E-24	UNDD	5 (1-5)	109
12	147	6.03E-21	DNUU	5 (1-5)	144
13	141	6.28E-21	DUD	4 (1-4)	408
14	45	1.45E-17	UDU	4 (1-4)	302
15	83	1.90E-16	NUNN	5 (1-5)	224
16	146	1.58E-11	DNU	4 (1-4)	415
17	42	3.30E-11	UNDN	5 (1-5)	131
18	148	6.00E-11	DNUN	5 (1-5)	192
19	40	7.60E-11	UND	4 (1-4)	298
20	106	1.04E-08	NDNN	4 (1-6)	664
21	159	1.37E-07	DDUU	5 (1-5)	56
22	79	4.41E-07	NUUN	5 (1-5)	97
23	81	7.53E-06	NUN	4 (1-4)	839
24	92	3.88E-05	NNUN	5 (1-5)	52
25	99	4.79E-05	NNDN	5 (1-5)	39
