# Supplementary material for: A polynomial time biclustering algorithm for finding approximate expression patterns in gene expression time series
Source: Algorithms Mol Biol. 2009 Jun 4;4:8. doi: 10.1186/1748-7188-4-8 (PMC2709627; doi:10.1186/1748-7188-4-8)
Supplement: Additional file 5 — Highly significant 1-CCC-Biclusters versus highly significant CCC-Biclusters. Table showing a comparison between the 47 highly significant 1-CCC-Biclusters discovered by 1-CCC-Biclustering restricted to errors in the 1-neighborhood of the symbols in the alphabet Σ = {D, N, U} and the 16 highly significant CCC-Biclusters found by CCC-Biclustering (after the applying the overlapping filter) and analyzed by Madeira et al. [9]. Both sets of biclusters were identified when the algorithm was applied to the DiscretizedHeatShock dataset. [file 1748-7188-4-8-S5.pdf]

Highly significant 1-CCC-Biclusters versus highly significant CCC-Biclusters

#	*ID*	*p-value*	*Pattern*	*Time Points*	*#Genes*	**Corresponding CCC-Biclusters**
1	10	0.00E-00	DDNU	1-5	1079	#1(ID 124) → Extended with genes and column at left<br>#8 (ID 151) and #21 (ID 159) → Extended with genes
2	27	0.00E-00	DNUU	1-5	597	#12(ID 147), #8(ID 151), #18(ID 148) and #21(ID 159)<br>→ Extended with genes
3	45	0.00E-00	DDU	2-5	1264	#1(ID 124) → Extended with genes
4	49	0.00E-00	DNU	2-5	1467	#1(ID 124) → Extended with genes
5	53	0.00E-00	DUU	2-5	1356	#1(ID 124) → Extended with genes
6	64	0.00E-00	NDDU	1-5	670	
7	79	0.00E-00	NNND	1-5	849	#5(ID 39) → Extended with genes
8	92	0.00E-00	NUND	1-5	1151	#3(ID 14) → Extended with genes and column at left<br>#4(ID 27) and #15(ID 83) → Extended with genes
9	132	0.00E-00	UNDD	1-5	539	#11(ID 43), #5(ID 39) and #17(ID 42)<br>→ Extended with genes
10	149	0.00E-00	UUND	1-5	1142	#3(ID 14) → Extended with genes and column at left<br>#4(ID 27) and #5(ID 39) → Extended with genes
11	161	0.00E-00	UND	2-5	1803	#3(ID 14) → Extended with genes
12	136	8.31E-42	UNND	1-5	665	#5(ID 39), #4(ID 27) and #11(ID 43)<br>→ Extended with genes
13	145	2.81E-41	UUDD	1-5	511	#4(ID 27) and #11(ID 43) → Extended with genes
14	157	6.05E-41	UDD	2-5	1538	#3(ID 14) → Extended with genes
15	165	1.04E-38	UUD	2-5	1577	#3(ID 14) → Extended with genes
16	14	1.36E-37	DDUU	1-5	521	#21(ID 159) and #12(ID 147) → Extended with genes
17	67	1.19E-35	NDNN	1-5	754	#15(ID 83) → Extended with genes
18	96	2.05E-35	NUUD	1-5	765	#22(ID 79) → Extended with genes
19	68	1.65E-33	NDNU	1-5	800	
20	48	1.28E-29	DNN	2-5	1298	#1(ID 124) → Extended with genes
21	72	5.34E-29	NDUU	1-5	646	#21(ID 159) → Extended with genes
22	105	2.09E-24	NND	2-5	1579	#3 (ID 14) → Extended with genes
23	26	6.43E-21	DNUN	1-5	452	#18(ID 148), #24(ID 92) and #12(ID 147)<br>→ Extended with genes
24	125	9.88E-20	UDNU	1-5	750	
25	19	1.45E-17	DNDU	1-5	614	#10(ID 142) and #8(ID 151) → Extended with genes
26	153	8.31E-17	UUUD	1-5	475	#4(ID 27) → Extended with genes
27	22	2.30E-16	DNNN	1-5	515	#8(ID 151) and #18(ID 148) → Extended with genes
28	97	4.15E-15	NUUN	1-5	385	#22(ID 79), #15 (ID 83) and #24 (ID 92)<br>→ Extended with genes
29	88	1.07E-14	NUDD	1-5	702	
30	6	1.20E-14	DDDU	1-5	427	
31	9	1.10E-11	DDNN	1-5	454	
32	61	4.72E-11	NDD	1-4	909	
33	131	3.27E-10	UND	1-4	749	
34	120	5.12E-10	UDDN	1-5	307	#17(ID 42) → Extended with genes
35	63	1.99E-09	NDDN	1-5	292	#25(ID 99) → Extended with genes
36	39	2.88E-09	DUUN	1-5	430	#18(ID 148) and #22(ID 79) → Extended with genes
37	24	1.21E-08	DNU	1-4	866	
38	148	2.27E-08	UUN	1-4	1434	
39	34	1.13E-07	DUND	1-5	936	
40	80	1.77E-07	NNNN	1-5	301	#15(ID 83), #24(ID 92) and #25(ID 99)<br>→ Extended with genes
41	69	4.02E-07	NDU	1-4	1191	
42	78	8.94E-07	NNN	1-4	1224	
43	122	9.16E-07	UDN	1-4	1462	
44	7	1.12E-06	DDN	1-4	1321	
45	91	1.23E-06	NUN	1-4	1335	
46	95	2.02E-06	NUU	1-4	1125	
47	75	3.62E-05	NNDD	1-5	198	#25(ID 99) and #8(ID 43) → Extended with genes
