# Supplementary material for: A polynomial time biclustering algorithm for finding approximate expression patterns in gene expression time series
Source: Algorithms Mol Biol. 2009 Jun 4;4:8. doi: 10.1186/1748-7188-4-8 (PMC2709627; doi:10.1186/1748-7188-4-8)
Supplement: Additional file 6 — GO terms enriched and transcriptional regulations of the top 16 CCC-Biclusters. Table showing a detailed analysis of the GO terms enriched and transcriptional regulations of the top 16 CCC-Biclusters discovered with CCC-Biclustering. When the set of genes in the CCC-Bicluster have more than 10 transcription factors or more than 10 GO terms enriched, only the top 10 of each are shown. We only show the GO terms passing the Bonferroni correction for multiple testing at either the 1% level (highly significant) or the 5% level (significant). The p-values marked with * only passed the test at the 5% level. The p-values presented in the table are without correction as it is common practice in the literature. [file 1748-7188-4-8-S6.pdf]

GO terms enriched and transcriptional regulations of the top 16 CCC-Biclusters

ID	*#Genes*	*TFs (Top 10)*	*%*	*GO Terms Enriched (Top 10)*	*DF*	*p-value (level)*
124	904	Yap1p	32.1	ribonucleoprotein complex biogenesis and assembly	31.10	5.62E-81(4)
		Sfp1p	29.6	ribosome biogenesis and assembly	27.91	1.76E-76(5)
		Met4p	24.5	organelle organization and biogenesis	43.06	3.08E-38(4)
		Rap1p	18.7	RNA processing	22.65	3.75E-37(6)
		Rpn4p	16.9	rRNA metabolic process	15.47	9.12E-36(6)
		Arr1p	14.5	RNA metabolic process	34.93	1.02E-33(5)
		Sok2p	11.7	rRNA processing	14.83	1.10E-33(6,7)
		Ifh1p	11.6	nucleobase, nucleoside, nucleotide and nucleic acid metabolic process	44.34	1.12E-33(4)
		Fhl1p	11.6	cellular component organization and biogenesis	56.78	3.69E-32(3)
		Ino4p	9.7	ribosomal large subunit biogenesis and assembly	6.54	1.29E-26(6,5)
14	1091	Yap1p	28.8	carbohydrate metabolic process	10.87	2.77E-23(4)
		Met4p	24.4	catabolic process	15.55	6.27E-21(3)
		Sok2p	19.5	cellular catabolic process	15.04	3.83E-20(4)
		Aft1p	18.5	generation of precursor metabolites and energy	8.85	4.15E-20(3)
		Hsf1p	17.1	cellular carbohydrate metabolic process	9.48	5.08E-19(5)
		Msn2p	16.7	response to stress	16.06	1.53E-18(3)
		Rpn4p	15.8	energy derivation by oxidation of organic compounds	6.95	7.57E-16(4)
		Msn4p	14.5	macromolecule catabolic process	11.88	3.33E-15(4)
		Arr1p	11.9	cellular macromolecule catabolic process	10.49	9.17E-13(5)
		Ino4p	10.6	energy reserve metabolic process	2.91	3.71E-12(5)
27	290	Met4p	27.7	carbohydrate metabolic process	13.64	1.04E-08(4)
		Yap1p	27.0	response to stress	19.32	1.53E-07(3)
		Sok2p	26.0	regulation of carbohydrate metabolic process	4.55	6.28E-07(5,4)
		Aft1p	24.9	energy reserve metabolic process	4.55	6.91E-06(5)
		Hsf1p	22.1	regulation of metabolic process	18.18	1.01E-05(4,3)
		Msn2p	19.4	cellular carbohydrate metabolic process	1023	1.23E-05(5)
		Rpn4p	17.3	regulation of biosynthetic process	5.68	1.24E-05(5,4)
		Msn4p	16.6	regulation of biological process	22.73	1.34E-05(3)
		Arr1p	15.2	cell communication	10.80	1.74E-05(3)*
		Ino4p	13.1	carbohydrate biosynthetic process	5.68	3.25E-05(5)*
39	258	Sok2p	27.3			
		Yap1p	21.9			
		Met4p	19.5			
		Arr1p	14.5			
		Hsf1p	14.1			
		Aft1p	14.1			
		Rpn4p	12.5			
		Msn2p	12.5			
		Nrg1p	11.7			
		Ino4p	11.7			
151	232	Yap1p	18.1	cellular component organization and biogenesis	57.89	3.14E-11(3)
		Swi4p	13.8	cell cycle	20.53	5.39E-10(3)
		Met4p	10.8	cell cycle process	20.00	5.85E-10(4,3)
		Mbp1p	10.3	nucleobase, nucleoside, nucleotide and nucleic acid metabolic process	43.16	8.64E-10(4)
		Sok2p	10.3	cell cycle phase	16.84	5.22E-09(5,4)
		Arr1p	9.5	mitotic cell cycle	13.68	3.58E-08(4)
		Ste12p	9.1	organelle organization and biogenesis	37.89	6.50E-08(4)
		Rpn4p	8.2	biopolymer metabolic process	52.63	7.40E-08(4)
		Aft1p	7.8	primary metabolic process	66.84	9.08E-07(3)
		Ino4p	7.3	cellular metabolic process	68.95	1.20E-06(3)
48	182	Sok2p	15.0			
		Yap1p	15.0			
		Arr1p	12.2			

		Met4p	11.7			
		Ste12p	11.1			
		Aft1p	10.0			
		Rpn4p	8.9			
		Nrg1p	8.9			
		Hap4p	8.9			
		Mga1p	7.8			
142	248	Yap1p	23.8	cellular component organization and biogenesis	57.89	3.14E-11(3)
		Met4p	13.3	transcription, DNA-dependent	18.95	5.58E-07(7,6)
		Sok2p	12.9	RNA biosynthetic process	18.95	6.11E-07(7,6)
		Ste12p	12.1	microtubule-based process	7.37	9.93E-07(6)
		Abf1p	10.1	transcription	19.47	1.42E-06(5)
		Arr1p	10.1	chromosome organization and biogenesis (sensu Eukaryota)	19.47	1.42E-06(6)
		Mbp1p	8.9	chromosome organization and biogenesis	19.47	1.75E-06(5)
		Yox1p	8.5	microtubule cytoskeleton organization and biogenesis	6.32	2.93E-06(7)
		Rpn4p	8.1	regulation of transcription, DNA-dependent	14.21	3.36E-06(8,7)
		Rap1p	7.7	regulation of transcription	14.74	4.35E-06(7,6)
		Cbf1p	7.7			
43	109	Sok2p	19.4	response to chemical stimulus	20.31	5.58E-05(3)*
		Yap1p	18.5			
		Yox1p	13.9			
		Ste12p	13.9			
		Met4p	13.0			
		Aft1p	13.0			
		Skn7p	12.0			
		Swi4p	12.0			
		Rpn4p	12.0			
		Arr1p	11.1			
		Rap1p	11.1			
		Tec1p	11.1			
147	144	Ste12p	16.0			
		Yap1p	16.0			
		Rap1p	13.2			
		Sok2p	12.5			
		Met4p	12.5			
		Swi4p	12.5			
		Leu3p	11.8			
		Rpn4p	11.1			
		Phd1p	10.4			
		Ino4p	9.7			
		Tec1p	9.7			
83	224	Yap1p	29.0	response to stress	16.56	6.23E-05(3)*
		Met4p	22.8			
		Sok2p	17.9			
		Hsf1p	17.0			
		Rpn4p	16.1			
		Aft1p	16.1			
		Arr1p	15.2			
		Ste12p	12.1			
		Msn2p	10.7			
		Rap1p	10.3			
42	131	Yap1p	22.1	cellular component organization and biogenesis	54.95	3.95E-05(3)*
		Aft1p	20.6			
		Sok2p	19.1			
		Hsf1p	18.3			
		Met4p	18.3			
		Ste12p	13.7			
		Msn2p	13.0			
		Phd1p	13.0			

		Arr1p	13.0			
		Msn4p	11.5			
148	192	Met4p	21.9	lipid metabolic process	11.49	2.41E-05(4)*
		Yap1p	20.8	cellular lipid metabolic process	10.81	4.45E-05(4,5)*
		Sok2p	15.6	protein modification process	17.57	6.48E-05(6)*
		Ste12p	13.0	ER to Golgi vesicle-mediated transport	6.08	7.22E-05(5-8)*
		Swi4p	11.5			
		Rap1p	11.5			
		Phd1p	9.9			
		Gcn4p	9.4			
		Rpn4p	9.4			
		Abf1p	9.4			
		Mbp1p	9.4			
159	56	Yap1p	33.9	cellular biosynthetic process	0.2500	1.93E-05(4)
		Met4p	17.9	regulation of translational initiation	6.82	3.36E-05(6-8)*
		Sfp1p	17.9			
		Cbf1p	14.3			
		Gcn4p	12.5			
		Yox1p	12.5			
		Pho4p	12.5			
		Leu3p	10.7			
		Rpn4p	10.7			
		Rap1p	10.7			
79	97	Yap1p	33.0	carboxylic acid metabolic process	19.72	4.75E-06(5)
		Met4p	22.7	organic acid metabolic process	19.72	4.75E-06(4)
		Sok2p	19.6	phosphatidylcholine biosynthetic process	4.23	4.24E-05(8-10)*
		Rpn4p	18.6	phosphatidylcholine metabolic process	4.23	9.96E-05(8,9)*
		Arr1p	17.5			
		Gcn4p	16.5			
		Hsf1p	13.4			
		Leu3p	11.3			
		Crz1p	11.3			
		Ste12p	11.3			
		Aft1p	11.3			
92	52	Yap1p	32.7	nitrogen compound metabolic process	28.57	4.55E-07(3)
		Sok2p	23.1	amine metabolic process	22.86	1.93E-05(4)
		Leu3p	19.2			
		Met4p	19.2			
		Rpn4p	15.4			
		Ino4p	15.4			
		Ste12p	15.4			
		Tec1p	13.5			
		Hap4p	13.5			
		Gcn4p	13.5			
99	39	Yap1p	28.2	organelle inheritance	13.79	2.58E-05(5)
		Ste12p	20.5			
		Sok2p	20.5			
		Met4p	17.9			
		Rap1p	15.4			
		Sfp1p	15.4			
		Rpn4p	12.8			
		Leu3p	12.8			
		Yox1p	12.8			
		Cin5p	12.8			
		Hsf1p	12.8			
		Ifh1p	12.8			
